# Strengthening the relationship between community resilience and health emergency communication: a systematic review

**DOI:** 10.1186/s44263-024-00112-y

**Published:** 2024-11-27

**Authors:** Tushna Vandrevala, Elizabeth Morrow, Tracey Coates, Richard Boulton, Alison F. Crawshaw, Emma O’Dwyer, Carrie Heitmeyer

**Affiliations:** 1 Centre for Applied, Health and Social Care Research, Faculty of Health, Science, Social Care and Education, Kingston University, Kingston Upon Thames, UK; 2 Independent Researcher & Expert in Residence Inclusive Research and Cultures, Kingston University, Kingston Upon Thames, UK; 3 School of Built Environment and Geography, Faculty of Engineering, Computing and the Environment, Kingston University, Kingston Upon Thames, UK; 4 School of Health and Medical Sciences, City St George’s, University of London, London, UK; 5 Institute for Infection and Immunity, St George’s, University of London, London, UK; 6 Institute for Lifecourse Development, University of Greenwich, London, UK; 7 Department of Anthropology, London, School of Economics and Political Science, London, UK

**Keywords:** Community resilience, Health emergency, Emergency communication, Vulnerability, Inclusive risk communication, Equitable recovery

## Abstract

**Background:**

Community resilience and health emergency communication are both crucial in promoting a community’s ability to endure crises and recover from emergency events. Yet, a notable gap in theory and evidence exists in the relationship between them. We aim to explore the relationship between community resilience and health emergency communication and to identify strategies and interventions to strengthen their usefulness to each other. Based on the results, a secondary aim was to develop a model of community-centred resilience and health emergency communication.

**Methods:**

A systematic review of literature published between January 1990 and February 2024 was undertaken following Joanna Briggs Institute guidelines. Electronic databases (Web of Science, Social Science Citation Index, PubMed/MEDLINE) were searched using key terms. Eligibility criteria were developed from the literature and the knowledge of the multidisciplinary team. Inductive thematic analysis generated key themes. The Preferred Reporting Items for Systematic Reviews and Meta-Analyses (PRISMA) guidelines were applied to present the findings.

**Results:**

The searches identified 300 articles, of which 86 met the inclusion criteria. Two main themes were identified from the literature: (i) the relationship between emergency communication and community resilience, including subthemes: building trust and collaboration within communities, identifying resources and their distribution, tailoring communication strategies, considering inclusion and equity, and community engagement and feedback and (ii) strategies and interventions, including subthemes: facilitating community structures as channels for communication, respecting personal and private boundaries in health communication, targeting outreach for effective crisis communication, building resilience through training and communication initiatives, and demonstrating commitment to equity and inclusion.

**Conclusions:**

There is a small, yet valuable, body of evidence to demonstrate the value of bolstering community-centred resilience for emergency preparedness, response and recovery. The model of community-centred resilience and health emergency communication developed can inform policy, research and practice. Further research is required to develop and test community-centred approaches to enhance inclusive risk communication and equitable recovery.

**Supplementary Information:**

The online version contains supplementary material available at 10.1186/s44263-024-00112-y.

## Background

Community resilience describes the collective strength, preparedness, and adaptive strategies of community members to minimise the impact of adverse events on a community and promote long-term wellbeing [1–3]. It encompasses a community’s ability to endure, adapt to, and recover from challenges such as natural hazards, economic hardships, or social crises [4–8]. Despite concerns about how community resilience may be operationalised and the potential for governments to shift their responsibilities without adequately supporting communities, this approach remains crucial for managing emergencies. Recently, the notion of ‘community-centred resilience’ has emerged in the face of criticisms about the limitations of top-down disaster planning [9]. This definition recognises that responsibility for responding to an emergency cannot wholly be devolved from government or agencies to community organisations. Partnership and engagement with existing community networks and organisations are essential for an effective response through collaboration rather than control. Nonetheless, there are many different ways of exploring community resilience. It has been perceived as being intricately connected to individual resilience, family resilience and business resilience, as well as the wider resilience of societies [4, 10–17].

Research shows that numerous social, cultural, economic, environmental and institutional factors shape a community’s resilience and capacity to withstand and recover from adverse events [1, 4, 18]. Previous authors highlight the crucial role of external factors, including access to resources and information, and emphasise that community resilience should not imply communities must manage entirely on their own. Internal factors within communities such as local knowledge, community structures, values, traditions, networks, coping strategies, grassroots efforts of community members and many other physical and cultural elements are known to contribute to community resilience [19–22]. Measures and indicators of community resilience show that resilience changes over time and varies between different communities [18, 22, 23].

The concept of 'community' provides a structure of meaning, which may or may not be linked to a certain place or locality, generating a shared understanding and basis for resilience behaviours [24]. Different communities in different countries tend to place different emphasis on various components of community resilience [25]. Authors have also described the chronic ‘weathering’ effects that some communities experience, where there is resilience erosion over time [26]. This is thought to contribute to the differential burden of acute emergencies on the resilience of disadvantaged communities [27].

In terms of what constitutes a 'health emergency', the World Economic Forum (WEF) Global Risks Report [28] and national risk registers, such as the United Kingdom’s (UK) Risk Register [29], identify diverse risks citizens face. These range from pandemics to natural hazards and cyber-attacks, each presenting distinct health challenges. Effective health communication about emergency events is crucial for community resilience as it empowers individuals with information and access to resources, fostering collective understanding, adaption or adoption of coping strategies [30–33]. Effective health emergency communication also supports public understanding and cooperation with advice, before and after emergencies, by employing a range of strategies such as risk communication and community engagement (RCCE) [34–42]. In﻿creasingly digital and social media communication are used to provide information about emergencies and recommended courses of action [43–45]. These strategies emphasise shared risk understanding, prevention, preparation, mitigating panic and confusion, fostering trust in authorities and bolstering confidence in healthcare systems [32, 38, 46–49].

Formal ‘health communicators’, such as government agencies and public service organisations and healthcare staff, have responsibilities to provide targeted and timely information to the public, to promote public health, enhance preparedness and guide appropriate responses during crises [32, 50]. For example, in the United Kingdom (UK) Local Resilience Forums (LRFs) are multi-agency partnerships comprising representatives from local public services, including emergency services, local authorities, the National Health Service (NHS) and the Environment Agency, known as category 1 responders [51]. Resilience-building initiatives emphasise a whole-of-society approach [52] where resilience is built ‘one community at a time’ [53]. During public health emergencies, such as the COVID-19 pandemic, the need for effective communication between agencies and communities is paramount for saving lives [54–56].

Traditional communication models often emphasise one-way, top-down dissemination of information from credible health authorities. Risk communication based on the public deficit model attributes failures in communication to inadequacies in the public’s understanding [37]. However, this approach overlooks the vital importance of upward communication within a two-way communication process—where communities provide critical feedback, express their needs and share local knowledge. New research argues that warnings are not just a siren or phone alert but should be a long-term social process that is a carefully crafted, integrated system of preparedness involving vulnerability analysis and reduction, hazard monitoring and forecasting, disaster risk assessment and communication [57]. Studies have demonstrated that effective communication strategies must be designed to be bi-directional, with communities and agencies engaging in a dialogue rather than hierarchical top-down dissemination [58–60].

This paper focuses on the relationship between community resilience and health emergency communication. The COVID-19 pandemic and other recent public health emergencies, such as the Ebola virus disease outbreak in West Africa (2014–2015), have highlighted significant challenges in health emergency communication [55]. These challenges include challenges for formal health communicators in understanding, engaging and effectively communicating with communities, especially those that are seldom heard or most at risk [61–63]. Intersectionality of risk factors is underexplored in the context of understanding vulnerability and needs [64, 65]. Vulnerability can be associated with chronic issues, such as longterm health conditions or poverty, in combination with the acute health risks created by emergencies. Identifying vulnerable groups also presents operational challenges, as people generally don't want to be labelled as being vulnerable and vulnerability varies with each emergency [1, 66, 67].

Neglecting active engagement with community leaders, members and networks in emergency preparedness can lead to adverse outcomes, such as scepticism towards vaccination and testing, exacerbating health inequalities [68–73]. Assumptions about communities can fuel misinformation and mistrust, underscoring the importance of targeted, community-centred interventions that include less visible and more vulnerable groups [34, 74–76]. Effective communication during emergencies relies not only on the dissemination of credible information but also on fostering genuine collaboration with the communities affected. Imposing solutions on communities without engaging them as equal partners in the response process can lead to ineffective outcomes and erode trust [77, 78]. Building partnerships that prioritise two-way communication ensures that the needs, concerns and local knowledge of communities are fully integrated into emergency responses. This paper examines how such collaboration can be achieved and the impact it has on health communication strategies during crises.

Previous studies have highlighted the critical role of community resilience in coping and adapting to health emergencies [79, 80]. However, limited research has specifically explored its intersection with health emergency communication or the notion of community-centred resilience. While some studies have attempted to conceptualise this relationship or bridge discipline-specific theories like The Communication Theory of Resilience and Discourse of Renewal [27, 81–83], there are no systematic literature reviews examining the relationship between community resilience and emergency health communication. Addressing this gap could offer valuable insights to tailor strategies effectively, fostering collaboration with communities rather than imposing solutions on them [84, 85]. It could also inform strategies for equitable resilience and recovery, where resilience practice takes into account issues of social vulnerability and differential access to power, knowledge and resources [86].

Communities with limited resources often struggle to coordinate communication efforts during emergencies, underscoring the importance of robust communication systems. Research indicates that communities with strong communication networks recover more effectively from disasters [4]. Therefore, integrating communication strategies into resilience-building initiatives could enhance community preparedness and response capabilities, contingent upon a local focus and capacity for implementation [32, 38, 46]. Furthermore, health disparities within and between communities intersect with specific health emergencies, impacting communities differently due to factors such as health, education, employment and communication differences [87, 88]. World Health Organization research demonstrates significant health outcome disparities among various groups, even in close proximity [89], with some communities facing additional barriers to accessing essential services due to low socioeconomic status, marginalisation and digital poverty [90].

The aim of this systematic review was to explore the relationship between community resilience and health emergency communication and to identify strategies and interventions to synergise their usefulness with each other. Based on the results, a secondary aim was to develop a model of community-centred resilience and health emergency communication.

The research questions were as follows:

- What is the evidence on the relationship between community resilience and emergency health communication?
- What interventions or strategies could strengthen the relationship between community resilience and emergency health communication?

This systematic review interrogates the evidence to gain important insights into the complexities of communities and their needs in relation to emergency communication. Both the community resilience and health emergency communication literatures recognise the diversity within communities, which can vary significantly in scale, size, geographical connections, faith, identity, shared experiences, digital engagement and affiliations. Each community embodies its own set of power dynamics, communication methods, language preferences, governance structures, norms, attitudes and historical contexts, resulting in distinct requirements and potential intra- or inter-community tensions or collaborations [34, 74–76]. Understanding these complexities could inform more targeted public health strategies, effective communication approaches, and bolster community resilience [32, 91].

## Methods

The systematic review followed the guidelines set out by the Joanna Briggs Institute (JBI) Manual for Evidence Synthesis [92]. The presentation of this systematic review follows PRISMA guidelines [93] using the PRISMA 2020 27-item checklist [94] (see Additional file 1). A search protocol was not developed or published. The search was registered with CABI Digital Library searchRxiv (accessible at https://www.cabidigitallibrary.org/doi/10.1079/searchRxiv.2024.00477). Electronic searches were performed for records between January 1990 and Feb 18, 2024.

### Sources

Electronic research databases Web of Science, Social Science Citation Index, and PubMed/MEDLINE were chosen as the most suitable sources as these databases have the widest international coverage of relevant interdisciplinary scholarly literature [95].

### Key search terms

Databases were searched using the search string developed from the key terms relating to 'community resilience' and 'health emergency communication' (see Additional file 2).

### Eligibility criteria

The eligibility of articles was determined according to defined inclusion and exclusion criteria. The criteria were developed from the literature, refined and agreed upon by the team in line with JBI guidelines for study selection and critical appraisal [96].

The inclusion criteria were as follows:

- Articles published in the research literature (journal articles, chapters/books, reports) examining community resilience and health communication within the context of acute health risks or emergencies.
- Provision of evidence or practical information concerning health communication.
- Published in the English language.
- Published on or after January 1, 1990 (to February 18, 2024).

**Fig. 1:**
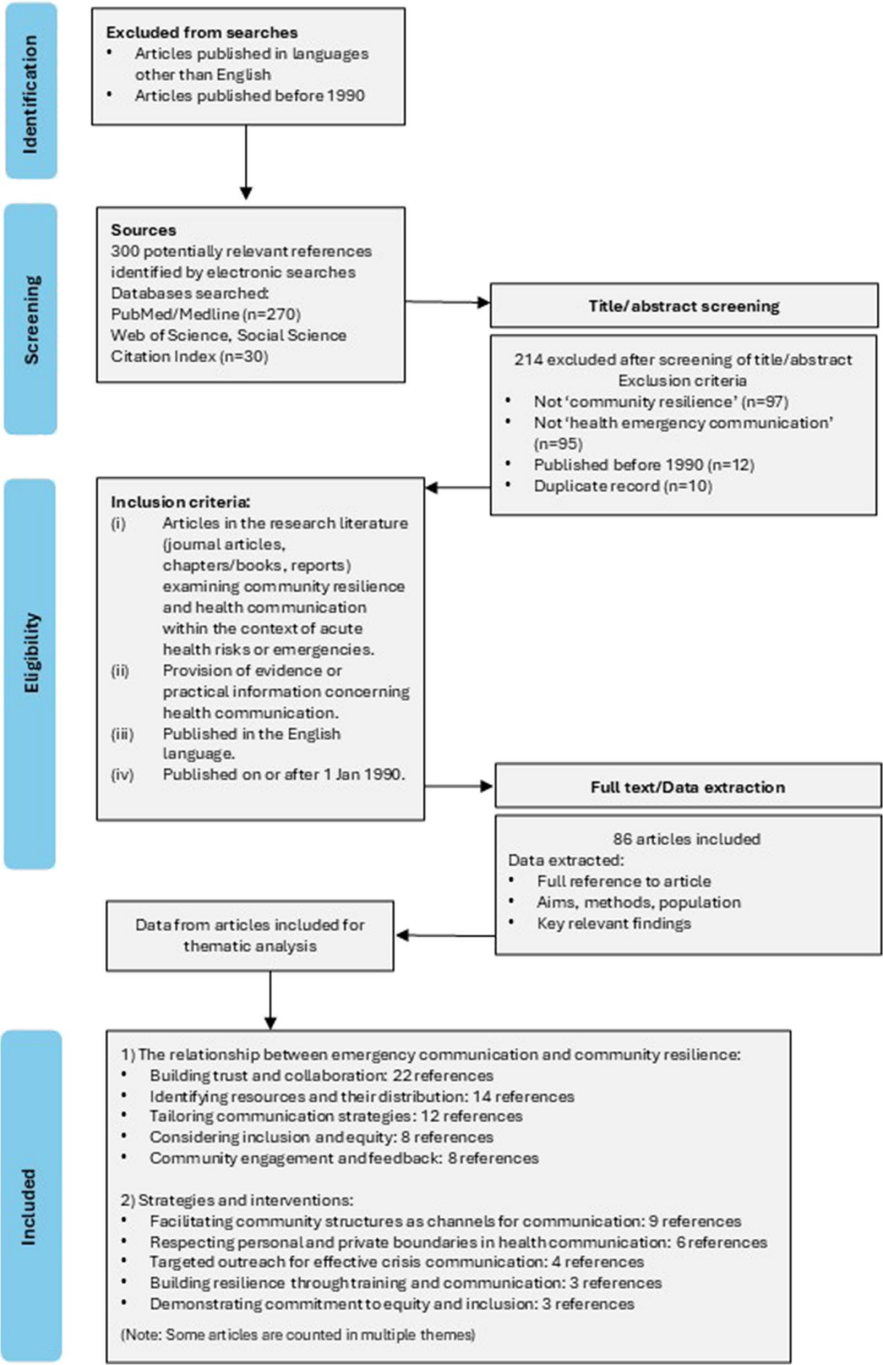
PRISMA flowchart

Details of the exclusion criteria are shown in the PRISMA flowchart (Fig. 1).

### Selection process

Articles were screened in Microsoft Excel by title and/or abstract. Included articles were checked by a second researcher against the eligibility criteria and level of evidence assessment (detail below). Duplicate records were counted and removed (detail in Fig. 1). A record of full references including electronic hyperlinks was created to enable article retrieval from the database. Full copies of included articles were retrieved from journal websites or repositories and downloaded for data extraction. No further articles were excluded at the full-review stage.

### Data charting

Data were extracted from relevant articles by one researcher (full reference to an article, aims, methods, population, key relevant findings) and were organised in literature tables in Microsoft Excel to facilitate familiarisation [97]. The data charting process ensured consistency so that extracted data retained links to original source documents for ease of retrieval. This systematic process helped to organise the data for analysis and sort and search within the data using specific key terms. The rigour of the data selection, coding and analysis was enhanced by a second researcher checking the included articles to validate the allocated thematic codes and GRADE (Grading of Recommendations, Assessment, Development and Evaluations) classification [98] (described below). The main outcomes of interest were the effects on processes or outcomes of community resilience and/or the influence or synergy with emergency communication as well as interventions or strategies for improving such outcomes. Variables of interest included contextual information (place, date, communities) and the types of emergency communication involved (recorded in the literature tables).

### Level of evidence assessment

An overall assessment of the body of evidence was made using the GRADE classification system [98]. This assessment system was selected as it takes into consideration a combination of study design and relevance of the results, rather than only focusing on one or the other. An assessment of the study design is made, as follows. Evidence from a systematic review of randomized controlled trials or individual randomised controlled trials is graded high (grade A). Evidence from a systematic review of cohort studies, individual cohort studies/low-quality randomised control studies, systematic review of case–control studies and individual case–control studies is rated medium (grade B). Evidence from case series, low-quality cohort or case–control studies is rated low (grade C). Expert opinions based on non-systematic reviews are classified as low (grade D). A second researcher independently reviewed 50% of the articles, and consensus on the final grading decision was reached following an online discussion between both researchers. In the present review, no papers were removed after grading and no weighting was applied to individual papers based on study design. Instead, the overall combined assessment of grades was used to gain an understanding of the combined evidence, heterogeneity of study type, and the level of evidence in relation to the specific questions of the review.

### Analysis

An inductive thematic analysis was undertaken to generate key themes regarding synergies between community resilience and emergency health communication (research question 1) and identify and select illustrative case studies of the relationship (research question 2) [99]. The initial familiarisation stage began with immersion in the raw data, reading abstracts and highlighting relevant extracts of text or figures from full articles. The analysis focused on allowing themes to be developed from the bottom-up (inductive approach). The first author and three co-authors met online to discuss prominent concepts and issues that the literature addresses, such as community engagement, equality and power dynamics, to understand how these issues fit together and to identify themes. Code categories were developed iteratively as the analysis went along [100]. The team discussed and identified biases in the data, including publication bias and methods bias (more detail is in the Discussion section). Case studies were selected on the basis of their potential to illustrate themes in the data and to demonstrate the range of study types and contexts in the topic area.

Two main overarching themes were generated. Once the themes were identified, these were compared with existing theories and concepts in the literature (see the Discussion section). This helped us confirm points of alignment with previous research, increasing confidence in the analysis. The data structure that was developed during the thematic analysis was then compiled into a conceptual model, presented in the discussion. This model encapsulates the findings and insights derived from the data and can inform future policy, research and practice [101].

## Results

The results section provides a brief summary of the included articles before explaining the two main themes generated from the data.

- The relationship between emergency communication and community resilience
- Strategies and interventions to enhance community resilience and health emergency communication (illustrated by selected case studies)

### Characteristics of the included articles

Of 300 potentially relevant articles identified by electronic searches and screened, 86 met the inclusion criteria and were included in the review. Details of the number of returns for each database and reasons for exclusions are in the PRISMA chart (Fig. 1). References and summaries of all included articles are shown in a supplementary bibliography (Additional file 3).

In terms of the overall level of evidence, the combined GRADE profile was as follows: only 1 article was grade A (1.1%), 30 articles were grade B (35%), 40 articles were grade C (46.5%), and 15 articles were grade D (17.5%). Thus, almost all of the evidence in this systematic review (98.9%) is medium to low grade (B–D). The GRADE classification given to each article is shown in the supplementary bibliography tables for transparency of reporting only, and it was not used to compare or weight the evidence from individual studies, which were all deemed to be relevant and useful for this systematic review.

The thematic analysis generated two main themes and ten subthemes as illustrated in Table 1. These are described below. The systematic approach gives a high level of certainty in the themes developed from the included literature, which can be traced back to the original articles.

**Table 1:** Overview of themes and subthemes in the literature

***Theme 1: The relationship between emergency communication and community resilience***	*No. of articles*
Building trust and collaboration within communities	22
Identifying resources and their distribution	14
Tailoring communication strategies	12
Considering inclusion and equity	8
Community engagement and feedback	8
***Theme 2: Strategies and interventions to enhance community resilience and health emergency communication (16 case studies)***	
Facilitating community structures as channels for communication	9
Respecting personal and private boundaries in health communication	6
Targeting outreach for effective crisis communication	4
Building resilience through training and communication initiatives	3
Demonstrating commitment to equity and inclusion in communication	3

The total number of articles exceeds 86 as some addressed more than one theme

### The relationship between emergency communication and community resilience

This section of the results presents evidence from the literature relating to the relationship between community resilience and health emergency communication, focusing on ways in which they interact.

### Building trust and collaboration within communities

The evidence suggests that trust and collaboration within communities are foundational elements of community resilience that can facilitate effective communication during health emergencies [102–104]. The paper by Imesha Dharmasena et al. [102] explores the role of public relations in building community resilience to disasters caused by natural hazards, offering perspectives from Sri Lanka and New Zealand. It highlights how trust-building efforts through strategic communication contribute to community cohesion and resilience. In addition, Marfori et al. [103] discuss public health messaging during extreme smoke events caused by wildfires in Tasmania, emphasising the importance of trust in disseminating health information effectively during crises. Miles et al. [104] illustrate how the frequency of emergency communication and knowing when to expect communication to occur can affect perceptions of community resilience in the context of natural hazards.

Community cohesion, also referred to as ‘groupness’, is also an important factor that affects how communities respond to various emergency communications [105]. The literature indicates that in times of recovery, community networks and connections, bonded by groupness, can support trust and collaboration, helping to manage the enduring effects on communities, such as mental health issues and the effects of longterm illnesses [106, 107]. Trust is built through the community’s relationships, addressing social identities, social norms, assets, values and traditions. Robert Punam’s idea of ‘social capital’ [108] has been used to describe these positive and productive aspects of sociability for community resilience [14, 15]. However, there is well-established literature that critiques the concept of social capital due to its potential to reinforce inequalities and support negative behaviours, as well as its conceptual ambiguity and measurement challenges [109, 110].

Community dynamics can foster or hinder inter and intra-community trust and collaboration, as well as perpetuating positive or negative behaviours and outcomes through ‘behavioural contagion’ [111]; however, this is a contested term in the psychology literature. Research suggests that interactions between social cohesion (groupness) and individual person characteristics can lead to conformity or exclusion [111]. How these factors affect trust and collaboration in relation to emergency communication is unclear. Understanding the gaps in the evidence on psychosocial-behavioural aspects of community resilience is essential for informing trust-building initiatives that achieve improved health outcomes [112]. For example, research conducted during the COVID-19 pandemic shows that phenomena that include—‘risk deliberation networks’, voluntary compliance with government guidelines and citizens’ subjective health experiences—influenced each citizen’s health-related behaviours and community-led risk discourses in the face of the urgent health crisis [54].

This literature elaborates on how various communities’ ‘social bonds’ (interpersonal connections), ‘cultural memory’ (or collective remembering) and historical background provide the foundations for trust and community cohesion [113]. A previous review of the literature found that maintaining cultural traditions post-disaster promotes community cohesion, upholding a sense of belonging and solidarity, which aids in psychological and social recovery [4]. Similarly, a study by Paton and Johnston [114] highlights how cultural identity acts as a trusted protective factor, mitigating the adverse effects of stress and trauma on community wellbeing. Other research focusing on indigenous communities facing climate crisis, demonstrates that acknowledging shared cultural context significantly enhances cooperative adaption in the face of environmental challenges [115]. In this example, the processes of ‘social learning’ and the development of shared mental models with communities, facilitates collective understanding and cooperation, strengthening community resilience in the face of climate emergency.

A recurring theme in the literature is the importance of different types of health communicators collaborating with communities rather than imposing top-down solutions. Successful emergency responses require not only disseminating information from credible sources but also engaging in partnerships where communities have a voice in shaping the communication strategies used. While some of the articles included in this systematic review hint at collaboration, this perspective was not consistently applied. For example, research by Jackson et al. [59] highlights the critical role of communities in co-designing emergency communication strategies, which contrasts with the more unidirectional approaches observed in many other studies. By examining the literature through this lens, it becomes clear that collaborative approaches are more effective in building trust, enhancing the dissemination of information, and ensuring that communication strategies are responsive to the real needs of communities. The literature on more collaborative forms of emergency communication also acknowledges that different communities and individuals are likely to want to collaborate in different ways, or not at all.

Across the literature overcoming historical mistrust and building genuine collaboration are recognised as key aspects of both community resilience and the success of health communication strategies [78, 116]. Community leaders, together with their leadership approaches, are known to play a vital role in this respect, helping to build trust and partnerships with different communities worldwide [117]. Evidence from several studies shows a diffused model of leadership across communities can create a resilient community by actively engaging members in addressing different types of health challenges [118, 119]. In the context of a health emergency, clarity about community leadership structures is essential for ensuring clear communication channels between the central government, local authorities and the community [44, 48].

A central perspective, regarding disaster prevention and management efforts, is to pivot health communication towards building communities’ understandings of acute risks and fostering trust [120, 121]. Clear communication procedures and reliable channels are considered crucial for timely and accurate information dissemination, reducing uncertainty and facilitating a coordinated effort to address immediate and long-term risks [49]. Looking across studies shows that while health communicators may seek to inspire confidence in authoritative preventive measures, resilient communities exhibit ‘adaptive capacity’ (as discussed by Klein et al. [122]), relying on trust and collaboration within their membership [123, 124]. Folke et al. highlight the importance of exploring these social dimensions of resilience, particularly in the context of the climate crisis, emphasising social processes like social learning and social memory, mental models and knowledge–system integration, visioning and scenario building, leadership, agents and actor groups, social networks, institutional and organisational inertia and change, adaptive capacity, transformability and systems of adaptive governance [125, 126].

### Identifying resources and their distribution

Being able to identify and access resources, such as information, assets, infrastructure and financial support, among communities are critical aspects of a community’s resilience [14, 127–130]. In terms of economic resources, there is robust evidence from many studies to show financial factors significantly shape community resilience, with stable economies enabling effective adaptation and communication [23, 131]. Resilient communities tend to have access to robust social infrastructure, including public buildings, shops, transport systems and telecommunication networks, while impoverished areas with high levels of social deprivation tend to lack such resources and the means to access them in emergencies or other times [130, 132].

Allocation of resources, such as targeted emergency preparation sessions in schools, provision or access to information communication technology (ICT) and social media communication can enhance both community resilience and effective health emergency communication [133–135]. Furthermore, provision of access to learning and information resources enhances community resilience by empowering individuals to adopt proactive measures during emergencies [80, 128]. Several studies internationally highlight the interrelated benefits of social and ecological resilience associated with equitable resource distribution for vulnerable populations during and after emergencies [15, 136, 137]. Fair resource distribution across various cities and counties has been found to promote trust, cooperation, inclusion and engagement, enhancing effective health communication in the face of health risks [32] and social vulnerability [127].

The role of community members and organisations in distributing resources and information is crucial for effective emergency responses. However, much of this work is unpaid or voluntary, or provided by already overstretched community organisations, which can exacerbate existing inequalities and place additional strain on communities [59, 138]. Community organisations, despite playing a key role in emergency responses, frequently struggle to secure the funding needed to maintain operations, let alone respond to crises. This evidence highlights a major gap in the current approach to community resilience in emergency contexts. Without proper funding, the burden on communities to fill resource and communication gaps risks further entrenching inequality and social deprivation.

### Tailoring communication strategies

In this literature, several studies have shown that insufficient insight into complex community dynamics, including cultural norms, shared identities and distinctive characteristics, coupled with a lack of understanding of preferred communication methods, has undermined health communication efforts [139–141]. For example, emergency messages may be perceived as culturally insensitive, untrustworthy or irrelevant to different communities. In the context of an 'infodemic' surrounding an emergency event such as the COVID-19 pandemic, ‘trusted messengers’ within communities can help to guide intended audiences to health information from credible sources [142]. To address these challenges, authors have emphasised the importance of co-designing and customising communication strategies with communities, not only to maximise the accessibility, effectiveness and resonance of health messages but also to ensure interventions are having the intended effects [143, 144]. Specific barriers to communication have been identified as including language, literacy levels and preferred information channels, however, their significance differs at the individual level, reflecting diverse communication needs and capacities across populations and places [144].

In the literature, tailored communication strategies are recognised as being crucial for effectively engaging often overlooked populations, such as school children’s understanding of natural disasters [145]. Digital poverty and digital literacy are significant barriers to communication in emergencies and the provision of ‘crisis informatics’ in preparation for emergencies [146–148]. Bukar et al. elaborate on social media’s role in COVID-19 recovery, underscoring the need for customised approaches to address different community’s primary concerns [147]. Tailored communication using culturally sensitive messaging is considered to be vital for effective health communication at various crisis levels and thresholds [4, 128, 148].

The literature also shows that actively involving communities in dissemination efforts can contribute to resilience by fostering a sense of ownership and empowerment among community members [16, 149]. It could be that involvement in dissemination enhances community cohesion and collaboration, enabling members to better identify and address their own unique contexts and challenges but this requires further research.

### Considering inclusion and equity

This literature suggests that communities emphasise inclusion and equitable benefits for their members, including access to resources and support [150–153]. Research has examined opportunities and challenges for public libraries to enhance community resilience, highlighting the role of libraries in providing equitable access to information and resources within communities [150, 151]. Furthermore, Sampugnaro and Santoro investigate the pandemic crisis and Italian municipalities’ responses, emphasising the need for inclusion strategies that address the diverse needs of communities during emergencies harnessing a ‘spirit of solidarity’ in the face of multiple endemic negative factors such as political fragmentation and poverty [152]. Review studies conducted by Norris et al. [4] and Patel et al. [154] provide evidence that communities with equitable access to healthcare services and strong mental health support demonstrate heightened resilience, possibly enabling recovery from emergencies. Additionally, research such as that conducted by Galea et al. [155] after the September 11 terrorist attacks in New York City, highlights the importance of a holistic focus on community inclusion for whole community healing and recovery following adversity. A community’s networks and bonds can ensure that during health emergencies, vital information and resources are shared and that all community members are included and informed about what to do [4, 154].

### Community engagement and feedback

While community feedback mechanisms are often referenced in health communication literature, the focus tends to remain on agencies as the primary drivers of communication. However, effective community engagement involves more than just receiving feedback, it requires ongoing, multi-directional communication where communities have a seat at the decision-making table. Grassroots communication enables communities to inform agencies about their specific needs, barriers to access, and unique cultural contexts, which are critical for tailoring effective health responses. As Poland et al. [9] and Jackson et al. [58, 59] argue, many communities are already motivated and willing to participate in emergency response efforts as equal partners, rather than being positioned as mere recipients of top-down information.

Research shows that the engagement of community members and the opportunity to provide feedback to health communicators, rather than simply the dissemination of standardised information, improves the effectiveness of health emergency communication [1, 152, 156–158]. In the context of COVID-19, community engagement in China and elsewhere helped to confront uncertainty and counter rumours effectively, strengthening international cooperation and evidence-based decision making for prevention and control measures [159]. Community resilience can support community engagement by disseminating timely and relevant information, for example via different communities’ school networks or social media networks [160, 161]. One example is the communication strategies of the US National Weather Service to protect communities, which emphasise the importance of community engagement in weather-related risk communication efforts. The approach is informed by the theory of ‘microboundary spanning’, where small-scale actions that connect different parts of an organisation or community, can foster collaboration and communication across boundaries [162]. Research on place-based communities that actively participate in decision-making, problem-solving and disaster preparedness further highlights a link between community engagement and effective emergency communication [163]. Involving communities in planning and response efforts, either through open community meetings or online forums, fosters a sense of community ownership and empowerment [1, 18].

The findings presented above show the ways that community resilience and health emergency communication complement each other. The second theme of the findings identifies strategies and interventions to enhance both community resilience and emergency health communication.

### Strategies and interventions to enhance community resilience and health emergency communication (case studies)

The findings in this section look more closely at the types of strategies and interventions that might enhance community resilience, with benefits for the effectiveness of health emergency communication. Table 2 summarises themes in the literature (drawing on 25 included articles) and provides 16 case studies from various countries and contexts, ranging from culturally inclusive strategies in emergency response, to recovery-focused peer health promotion projects in shelters, and government-funded community programmes addressing inequalities.

**Table 2:** Case studies illustrating strategies for community-centred resilience and health emergency communication

Strategies	Suggestions for actions based on case studies	Case Studies
**1. Facilitating community structures as channels for communication**	• Creating a **contact list, register or network** of active community members to distribute health emergency information	• The US Centre for Disease Control and Prevention’s (CDCP) “Access and Functional Needs Toolkit: Integrating a Community Partner Network to Inform Risk Communication Strategies” [164] has been developed to support health communicators to build partnerships and engage in meaningful communication with communities. The toolkit highlights the communication needs of diverse communities including children, pregnant women, older people, people with limited literacy or English language, people with limited transportation, people with disabilities, people with chronic; and provides tips for engaging partners and dissemination pathways and sustaining networks
	• Launching **targeted social media campaigns** to involve the public in resilience planning and communication efforts by utilising existing online platforms and online communities to engage a broader audience, gather feedback and disseminate information in an accessible and inclusive manner	• An international team of health communication researchers have developed a novel precision public health campaign framework [91] to structure and standardise the process of designing, developing and delivering tailored health messages to target particular population segments using social media–targeted advertising tools. The framework consists of five stages: defining a campaign goal, priority audience and evaluation metrics; splitting the target audience into smaller segments; tailoring the message for each segment and conducting a pilot test; running the health campaign formally; and evaluating the performance of the campaigns. The precision public health campaign framework has the potential to support higher population uptake and engagement rates by encouraging a more standardised, concise, efficient and targeted approach to public health campaign development
	• Encourage communities to develop **digital skills for social medial engagement**, and to develop the skills to be able to critique different sources of digital information	• During the COVID-19 pandemic US community members’ social media engagement was significantly associated with their perceived community resilience [165]. While helping others on social media led people to perceive their communities as less resilient, the use of social media for social support helped foster social capital, leading to more perceived resilience at the collective level. Overall, social media use played important roles in shaping people's perception of community resilience, helping community members and organisations evaluate their strengths and weaknesses, and make improvement to better address future challenges in the times of global disasters
**2. Respecting personal data and private boundaries in health communication**	• Organise **community forums** or dialogues focused on health-related topics, creating safe spaces for open discussions to encourage individuals to share experiences, concerns, and perspectives, promoting a sense of community understanding and support	• In 2019, The New York Public Library launched a pilot series in select library branches across three boroughs. “Community Conversations” encourages open dialogue where everyone’s voice can be heard [175]. Health dialogues using Community Conversations Café serves as a platform where the scientific and non-scientific communities can both gain insight and perspective. The goal of the programme series is for both groups to learn from each other through an organic, informed and respectful exchange. These programs are meant to inform participants in a meaningful and constructive way, discuss current scientific endeavours, shed light on the unknown, and dispel misinformation or fear
	• Establish **peer support networks** within communities, where individuals can connect with peers facing similar health-related challenges to facilitate interpersonal connections, reduce stigma, and provide a supportive environment for sharing thoughts and experiences	• In Norway, a recovery-oriented Internet-based portal called ReConnect was used by service users in two mental health communities for 6–12 months [176]. The portal included an online peer support group which also facilitated participation in local offline peer support groups. Both group formats were moderated by an employed service user consultant. Online and offline peer support groups complement each other, and that combining them was mainly described as beneficial by service users
	• Implement **educational programmes** that emphasise the interconnectedness of individual and community wellbeing to encourage a shift from exclusive focus on personal coping to understanding the role of collective resilience in maintaining community health	• Schools play a crucial role in providing safety and mental health assessments and interventions for children, especially in the context of disasters and crises, making them a critical setting for the delivery of health communications and resilience building interventions. Tailored, school-based interventions in Gaza [177] have proven effective in providing educational and psychosocial support for conflict-affected youths, addressing issues such as academic underachievement and its impact on mental health
	• Train community members as **health ambassadors** to serve as approachable figures for discussions around specific health concerns, to bridge the gap between formal health communication and community members, fostering trust and open dialogue about health issues and risks	• A wide range of health ambassador schemes at one English NHS trust in Bradford [178] include pathways to Action–training for community health activists, ‘Healthwise’ tutors running groups around physical activity and health topics, health apprenticeship schemes, health trainers, breastfeeding peer support, Asian outreach workers, buddy schemes around HIV/AIDS, voluntary walk leaders, community coaches, lay workers running food co-ops & cook and eat sessions, healthy living centres, sure start initiatives/children’s centres
	• Create **diverse communication channels**, including online platforms, helplines and community meetings, to cater to various preferences and ensure that health information reaches individuals through channels they are comfortable with, promoting engagement	• American Hospital Association [179] web-based information (tip sheet, key considerations and reflection questions) on communication strategies for public health emergencies emphasises health care leaders must be prepared for increasing threats to public safety. In emergency situations, informed decision-making relies on timely, accurate and coordinated communications. Part of an effective strategy is to use diverse communication channels to reach different communities so there is not an over reliance on any one mechanism
**3. Targeting outreach for effective crisis communication**	• Establishing **decision-making forums or platforms** for active community participation in decision-making processes related to resilience planning, empowering community members to contribute to and shape initiatives, ensuring cultural relevance and increasing community engagement	• The Council of Europe [180] have reviewed different forms of young people’s participation in decision making. Drawing on various international case studies, the key findings are that co-management, co-production, digital participation, deliberative participation and for some, the concept of ‘participatory spaces’ are seen as the more innovative forms of participation. Recommendations centre on establish a strategic approach to promoting youth participation practice. Strategies should be developed with all young people, including those from minority and disadvantaged groups, and encompass a broad definition of what constitutes youth participation in decision making, encompassing a myriad of forms for involving young people in decisions about all matters that affect them
	• Conducting **interactive emergency preparedness workshops** within communities to educate residents about emergency preparedness and response in order to foster a sense of community ownership, encourage dialogue, and empower individuals to actively participate in resilience-building efforts	• The CREATE Resilience project [181] centered on co-creating a community vision of resilience, specifically as it relates to natural hazards and climate change by focusing on a positive narrative. By engaging youth, artists, municipal officials and community members in a variety of activities, including surveys, story-gathering and photovoice exhibits, forums, artist-created murals, and ripple effect mapping (REM), the project increased knowledge of weather and climate, risks from local hazards, and strategies for mitigation, while leading the community in thinking about what resilience means
**4. Building resilience through training and communication initiatives**	• Implementing **cultural humility training** programmes for emergency responders, healthcare professionals, and community leaders to enhance understanding of diverse cultures, promote sensitivity, and improve communication before and during emergencies	• In 2012, Seattle Fire Department implemented a culturally inclusive strategy [183] including cultural awareness training programme for its emergency responders to better serve the diverse population in the city. The programme included workshops, simulations and ongoing education to enhance understanding of cultural nuances. This initiative resulted in improved communication during emergencies, increased trust within communities, and a more effective response to incidents in culturally diverse neighbourhoods
	• Training and deploying **community health ambassadors** who can act as bridges between official channels and diverse community groups to facilitate culturally sensitive communication, address community-specific concerns, and enhance trust in health initiatives	• Toronto Shelter Networks Peer Health Promotion Project [185] focuses on health promotion across many sites including the shelter sector, respites, 24 hour women’s drop-in, and COVID hotel programme sites. TSN Community Health Ambassadors are engaged in activities which include providing education and raising awareness to peers about disease prevention and health improvement, speaking with peers to address fears, questions and misconceptions and working alongside shelter staff and Toronto Shelter Network to plan and provide health promotion and education at their shelter sites
	• Creation and training of **community champions** for community-based outreach activities with a focus on inclusion of at-risk communities	• During the COVID-19 pandemic, the UK government set up a community champions funding award scheme for local authorities to develop local programmes that addressed emerging inequalities [186]. All local programmes aimed to reduce health inequalities by involving at-risk communities in local prevention efforts, adapting the approach to local priorities. Two levels of community engagement were volunteer mobilisation and subsequent community-based outreach activities. Elements of capacity building, such as training and creation of networks, were common. Stronger relationships with communities were regarded as a key mechanism to support more equitable prevention strategies. Local Authorities handed over the design of schemes to people who knew their neighbourhoods and their needs best. This enabled community champions to innovate in response to the immediate situation rather than audit goals introduced from the top down. This resulted in more diverse CCs and third sector organisations that were willing to work with the council when it did not prescribe the terms of engagement
	• Conduct **place-based round tables or consultation workshops** that bring together individuals and community leaders to explore and discuss resilience-building strategies collectively, identify community assets, foster collaboration, mutual understanding, and a shared commitment to building resilience within the community	• Led by Anglesey Council in Wales, a community resilience model was developed [60] based on a citizen-centred approach and asset-based community development principles; with the aims to create a shift in the balance of power between service providers and service users and involving local people in the decision-making. Delivery involved a 12-week period of consultation (including public meetings, mapping activities, informal chats with local residents, community groups and local organisations). Emphasis was placed on engaging seldom heard communities in the process. Results from the consultation were used to develop community-based initiatives addressing issues such as low levels of health and wellbeing, loneliness, and social isolation
**4. Demonstrating commitment to equity and inclusion in health emergency communications**	• Develop **targeted information campaigns** addressing sensitive health topics, emphasising the importance of open communication to increase awareness, reduce stigma, and encourage individuals to feel more comfortable discussing health-related matters within the community	• In emergency situations the World Health Organisation’s Incident Management System (IMS) [117] helps to expedite the development of technically sound and timely guidance and interventions to reduce health risks. As needed, WHO will deploy the WHO Emergency Communications Network (ECN)—a network of health communicators who undergone intensive pre-deployment trainings and have been certified to have the right skills to join response teams whenever needed
	• Developing and disseminating **emergency communication campaigns** in multiple languages and a range of formats to ensure that crucial information reaches all sectors of the population, overcoming communication barriers and promoting inclusivity	• The WHO’s Strategic Communications Framework for effective communications [117] aims to broaden reach to diverse audiences and ensure that health information reaches the people who need it. The framework includes processes and tools, with a commitment to publish in six languages. Multilingual content makes access to health information and WHO communication resources more equitable and effective

### Facilitating community structures as channels for communication

Facilitating community structures as channels for communication involves various strategies to ensure effective dissemination of health emergency information and engagement with diverse community groups. Establishing contact with networks of active community members and individuals with access and functional needs (e.g., individuals with and without disabilities, who may need additional assistance because of any condition, temporary or permanent, that may limit their ability to act in an emergency) can enable the swift distribution of critical updates [164] (case study: US Centre for Disease Control and Prevention). Targeted social media campaigns play a crucial role in involving the public in resilience planning and communication efforts, leveraging existing online platforms to engage broader audiences and disseminate information [91]; however, these benefits may not be accessible to all (case study: Precision public health campaign). Social media engagement has proven instrumental in shaping community resilience perceptions during the COVID-19 pandemic, with platforms facilitating social support networks and aiding in the evaluation of community strengths and weaknesses [165] (case study: United States COVID-19 social media engagement).

Other types of community structures, notably mutual aid groups and community grassroots support organisations have been shown to provide a pivotal role in community resilience [73, 166, 167]. Interventions to recruit and train ‘community health ambassadors’ or ‘community champions’ emerge as key drivers of resilience promotion, inspiring collective action and ensuring inclusivity by representing diverse voices within communities [168]. In Sri Lanka, mothers’ support groups played a pivotal role in empowering communities amid the COVID-19 pandemic: key contributions included establishing communication networks, fostering a supportive environment for preventive behaviours, organising vaccination clinics, distributing essential supplies, arranging recreational activities, promoting home gardening, and monitoring community activities [169].

Additionally, unpaid or family caregiver networks and formal community care networks (e.g., home carers, community support workers) serve as crucial communication channels for clinically vulnerable groups, reaching behind closed doors to those who may not be well enough or have the capacity to engage with health messaging systems [170, 171]. This raises the question of how to engage with, involve and support paid and unpaid carer groups in health emergency planning and better utilise their networks in emergency response efforts.

### Respecting personal data and private boundaries in health emergency communication

Research during COVID-19 illustrates that health communication often navigates sensitive personal and private territories, where individuals may be reluctant to disclose health information or openly discuss personal views or information, like vaccination status [69]. Challenges can arise when some community members choose not to engage, preferring to maintain distance or privacy, potentially as a coping mechanism against stigma or criticism [172]. An individual focus, on self-reliance and self-protection to cope with fear and stress, could impede communal resilience efforts by undermining community cohesion [173]. Thus, balancing individual privacy with fostering prosocial community-focused resilience is essential for a whole of society’s approach to health emergencies. The complexity of these issues is illuminated by trauma-informed community resilience models, which emphasise addressing resilience comprehensively at both individual and group levels, acknowledging the connections between personal coping strategies and community support systems [173, 174]. Initiatives such as organising community forums, establishing peer support networks and implementing targeted information campaigns can create safe spaces for open dialogue, reduce stigma and foster community understanding of individual and community health risks (case studies: New York Public Library [175], Norway ReConnect [176] and WHO Incident Management System [117]).

Educational programmes and school-based interventions can further promote collective resilience by emphasising the interconnectedness of individual and community wellbeing, underlining the critical role of schools in delivering health communication and resilience-building interventions [160] (case study: school-based interventions in Gaza [177]). This prompts the question of how communication initiatives can effectively uphold personal privacy while strengthening community resilience in the face of health emergencies (case studies: English NHS health ambassadors [178], American Hospital Association (AHA) web-based information [179]).

### Targeting outreach for effective crisis communication

This literature emphasises that targeted outreach is crucial for effective crisis communication and can be achieved through initiatives that encourage proactive community engagement in crisis communication. For example, establishing decision-making forums or platforms for active community participation has been shown to empower young people across Europe to contribute to resilience planning, ensuring cultural relevance and increasing community engagement (case study: Council of Europe young people’s participation [180]). Interactive emergency preparedness workshops have been used to educate residents about response strategies for natural hazards, fostering community ownership and dialogue while empowering individuals to develop community-centric definitions of resilience and participate in resilience-building efforts (case study: CREATE Resilience project [181]).

Tailoring outreach strategies to address specific community’s needs helps mitigate vulnerabilities, ensuring equitable distribution of support and resources, thereby bolstering economic recovery and overall resilience [149]. For example, in the context of earthquake risk, audience segmentation approaches, based on individual’s behavioural patterns, can engage different groups of the public more effectively than standardised national campaigns [182]. Thus, a range of targeted outreach strategies and interventions is required to effectively support the adaptable emergency responses that diverse individuals and communities typically need and prefer.

### Building resilience through training and communication initiatives

The literature indicates that building resilience through communication initiatives can help to develop an understanding of cultural dynamics, emphasising cultural literacy and humility as integral components (case study: Seattle Fire Department [183]). For example, Lekas et al. [184] advocate for an inclusive approach rooted in self-reflection, appreciation of lay expertise, power sharing and continuous learning, ensuring that resilience planning considers the diverse cultures within communities. Actively involving community members in decision-making processes can empower some members to shape initiatives’ cultural relevance, thereby enhancing their effectiveness and long-term sustainability [4].

Trends in the governance of health systems towards increasing active patient and public involvement (PPI) mirrored in public involvement in health research, further underscore the benefits of involving wider groups of the public in roles in emergency communication, such as the role of community health ambassadors (case study: Toronto Shelter Networks [185]). Other initiatives, such as training community champions [186] (case study: UK COVID-19 pandemic), community health champions, peer supporters or patient advocates can involve individuals already experienced and engaged in health systems or voluntary and community organisations (VCOs). Additionally, crisis communication strategies such as place-based roundtables or consultation workshops, which discuss the unique uncertainties that arise during emergencies, can enhance the potential of health communication to build community resilience (case study: Anglesey Council Wales [60]).

### Demonstrating commitment to equity and inclusion in health emergency communication

Upholding equality laws and anti-discrimination policies within social systems lays the foundation for an inclusive health emergency environment, to ensure that all communities receive crucial information and support during times of crisis [128, 137]. Developing and disseminating emergency communication campaigns in multiple languages spoken within the community is a crucial first step in overcoming language barriers and promoting inclusivity [117]. The WHO has developed a framework to broaden its reach to diverse audiences, committing to publish in six languages, thereby making access to health information and WHO communication resources more equitable and effective (case study: WHO [117]). By prioritising multilingual content and inclusive communication strategies (e.g., for those who are blind, partially sighted, D/deaf, hard of hearing or those with learning disabilities), health emergency communication could better serve the diverse needs of communities, promoting equity and inclusion in disaster response efforts. Furthermore, using evaluation methods that consider diverse perspectives can enhance the effectiveness of resilience initiatives to be assessed inclusively and developed more coherently to reflect the needs of diverse communities [128]. Another approach is to train health ambassadors to serve as approachable figures for discussions around specific health concerns within communities, building trust before emergencies occur (case study: Bradford NHS Trust [178]).

## Discussion

**Fig. 2:**
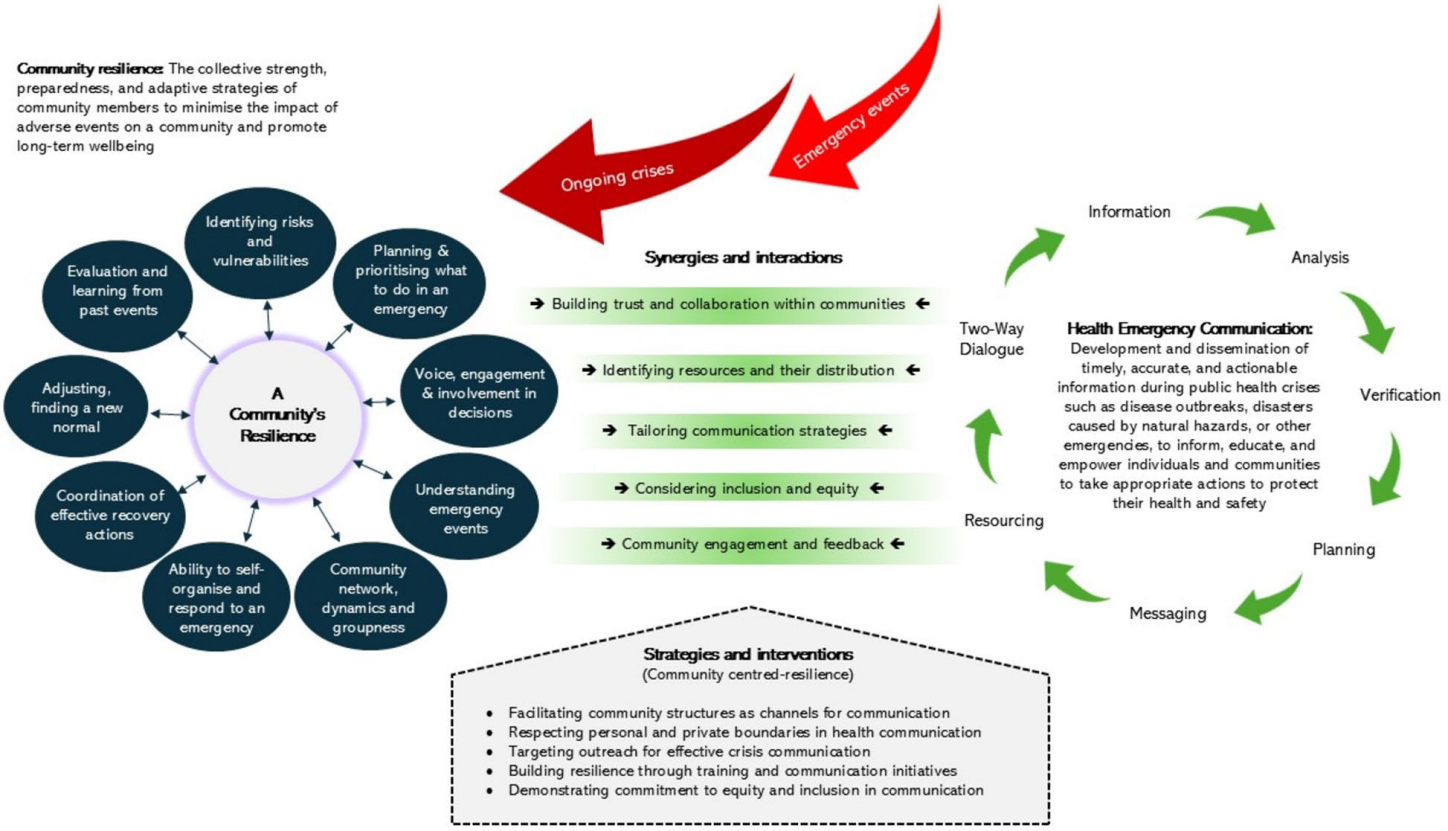
Model of community-centred resilience and health emergency communication

The next step in this systematic review was to draw together the themes in the literature into a conceptual model that represents the findings in an accessible visual representation. The model illustrated by Fig. 2 highlights the synergy between community resilience and emergency communication, adding a new perspective to existing conceptualisations [33, 117, 187]. This emphasis on synergy indicates ways that these two complex fields might be enhanced by strategies and interventions at their intersection, for the benefit of community-centred resilience. Future research could interrogate the fit of the model with existing models of community disaster risk management [156, 157], communication in community resilience [27], inclusive community preparedness programmes [188], empowerment of high-risk groups [124], collaborative action within and between communities [189], and trauma-informed models [173, 174].

Applying this practical model to the evidence highlights a number of interrelated implications for policy, research and practice. First, trust and collaboration with and within communities arise as important themes, confirming previous findings [77, 117]. A narrow view of the diverse meanings of the community may overlook inherent power dynamics and communication hierarchies that affect trust. Assumptions may also be made about the influence of prominent community leaders, overlooking less visible forms of group leadership and networks within communities. These issues could be examined further by drawing on ‘communities of practice’ theory which suggests that anyone within the community can contribute and influence direction and outcomes [119].

Second, the value of community resilience for identifying and mobilising resources equitably, between different members and between different communities, should be explored further through future qualitative, participatory or community-led research. Drawing on notions of mapping community assets and social infrastructure could be beneficial for exploring the meaning of resources in specific contexts [190]. An important yet underexplored issue that emerged from this systematic review is reliance on community organisations and volunteer members to disseminate information and resources during emergencies. This reliance, often without adequate financial or material support, can place an unsustainable burden on community organisations to recruit and sustain volunteer efforts. As Jackson et al. [59] and Kelly et al. [138] argue, the expectation that community organisations, networks or groups can bear the brunt of response efforts, particularly through volunteer labour, can exacerbate poverty and inequality. The evidence consistently underscores the nuanced interplay of community resilience with broader factors—such as economic stability, social infrastructure, education, health and wellbeing, and environment—to ensure effective and inclusive communication efforts [128, 136, 137]. Providing sustained funding for community organisations, both before and during emergencies, is crucial for enabling them to continue serving their communities and strengthen the resilience of community networks and groups, to ensure that they are well-prepared to mobilise to manage the distribution of resources and information in times of crisis.

Third, the model developed indicates that accessing community networks can help to achieve tailored communication strategies whilst promoting community resilience, which offers avenues for future research to examine the challenges and solutions of how to reach marginalised groups, within and between different communities and geographical places [191]. Several articles in this systematic review hinted at the value of grassroots community development as a strategy for building resilience. These studies pointed to the importance of empowering communities to create and sustain their own support networks, which are often better suited to addressing local needs than external agencies. However, future research is needed to examine how investment in community development may build resilience in vulnerable groups in low-income areas.

Fourth, in the relationship between community resilience and emergency health communication, the intertwined principles of inclusion and equity emerge as pivotal factors in the model that has been developed (Fig. 2). It is essential to recognise that these principles work synergistically, reinforcing each other’s importance. Looking forward, research could interrogate how community dynamics either foster or hinder equitable resilience and inclusive communication [86]. Such research endeavours hold the potential to deepen our understanding of how individual characteristics intersect with community dynamics and influence the effectiveness of specific health emergency communications [87, 88].

Fifth, strategies for community engagement and feedback can be developed and enhanced with communities to provide a more transparent and systematic approach to health emergency communication that takes into consideration how different communities prefer to engage and feedback to health communicators [40, 107], and the spaces where they prefer to do this, such as public libraries [151]. Future research, policy and practice in this area should give due consideration to the delicate balance to be struck between utilising community resilience and not burdening communities with excessive responsibility for health emergency communication, as well as providing community organisations with adequate compensation for emergency communication work.

Sixth, further research is needed to examine how community resilience is enacted or operationalised during specific emergency communications. For example, applying the model developed to ask, who is engaged in monitoring, how is risk monitored, how are community and their leadership identified, how is data collected and monitored in real time across a range of different stakeholders, and how are lessons from previous emergencies used to plan for future emergencies. Future research should also recognise that specific communities develop their own community-led unique approaches, models, and interventions to understand and build their community’s resilience, which may or may not be in conflict with the goals of agencies or top-down external interventions [192].

Finally, further research is needed to investigate the challenges and unintended consequences arising from some communities being more able to self-mobilise and adapt compared to others. For equitable recovery, it is vital to find ways to avoid “the creation of new inequalities” [193] by establishing ways to identify and support less able or vulnerable communities. Additionally, important questions remain about how the marginalisation of individuals or groups, within and between communities, may affect emergency and crisis communications and how to support communities to ask “how resilient are we?” There is a wealth of research on the topics of community advocacy and collective action that it has not been possible to include in this systematic review and future research could seek to integrate this evidence into the model developed.

The main strength of this systematic review is that it elaborates on the relationship between community resilience and health emergency communication, emphasising their dynamic interaction and mutual reinforcement, illustrating these findings in a new conceptual model. The results reveal a valuable body of evidence to demonstrate the synergies and interactions between community resilience and health emergency communication. Further empirical inquiry is essential to discern the optimal blend of interventions for the greatest benefit and impact in relation to different communities. 

It is essential to further explore the complexity and dimensions of the issues identified such as economic perspectives, community engagement, indicators of beneficial synergies, education and training needs, and implementation and sustainability with different stakeholders and communities. Additionally, broader power differences within society—such as disparities in agency, potential, resources and voice—impact communities’ ability to cope with disasters and the resonance of formal communication systems. By advancing this agenda collaboratively at a local level, nations can navigate future crises more adeptly, thereby fulfilling the whole-of-society approach one community at a time. A notable limitation of the studies reviewed is the relative underrepresentation of two-way communication approaches or partnerships between agencies and communities. Much of the literature focuses on top-down communication, which can undermine the value of community input and localised knowledge. For example, during the COVID-19 pandemic, many agencies struggled to communicate effectively with vulnerable groups, such as the homeless and isolated seniors due to a lack of established communication channels that would allow these individuals to express their needs [59]. Future research should place greater emphasis on mechanisms that facilitate upward communication from communities to agencies, ensuring that health communication strategies are co-produced and responsive to the lived realities of all members of society. Future research should also explore how best to implement and sustain these collaborative partnerships, particularly in the context of emergency response. The current literature hints at the value of these partnerships but often falls short of demonstrating their full potential. There is an urgent need for research that focuses on how two-way communication models, grounded in partnership, can be embedded in emergency planning and response efforts.

A key finding of this systematic review is the inconsistent focus on collaboration with communities in the studies examined. While some articles recognised the value of community partnership, many still adopted a top-down approach, focusing on dissemination of information from central agencies without fully involving communities in the decision-making process. This gap highlights the need for a more consistent approach to engaging community organisations and representatives as equal partners in emergency communication efforts. As noted in Jackson et al. [59], collaboration leads to better outcomes by ensuring that communication strategies are tailored to the specific needs and circumstances of each community, rather than imposing one-size-fits-all solutions.

A common assumption in the literature is that communities with lower socioeconomic status (SES) may be less resilient in the face of emergencies. However, evidence from community-level responses, such as those observed in Toronto during the COVID-19 pandemic [59], challenges this assumption. Even in disorganised or resource-poor communities, residents often find ways to support one another, particularly when informal networks or local organisations are present. Resilience, in this context, is not solely determined by SES but is instead deeply tied to the presence of community connections and the ability of individuals to mobilise collectively. This finding suggests that investing in community development and physical and social infrastructure, rather than relying solely on top-down government or agency interventions, is essential for enhancing community resilience. Grassroots-level support for building community networks and strengthening local organisations can enable communities to respond more effectively to emergencies. This investment not only fosters resilience but also ensures that communities can operate independently of external agencies when necessary, enhancing their ability to organise and respond to local needs quickly in times of crisis.

The primary limitation of the systematic review method can be over reliance on a quantitatively oriented systematic review process, which is often geared towards assessing studies with more robust experimental designs, such as randomised controlled trials (RCTs). In the context of emergency response, it is not surprising that only one article in our review received an ‘A’ grade under the GRADE assessment criteria. The ethical and practical challenges of conducting RCTs in emergency situations are well recognised in the literature. Our systematic review has highlighted that many high-quality community focused studies—particularly qualitative research—add value to the evidence-base. The use of case studies, qualitative studies, participative research and community-led research is essential for understanding the lived experiences, social dynamics and context-specific factors that influence real-world emergency response efforts.

## Conclusions

This systematic review elaborates on the relationship between community resilience and health emergency communication. It reveals how effective communication strategies bolster community-centred resilience and how resilient communities facilitate the dissemination and response to emergency messages. The role of community engagement in health communication should not be limited to disseminating information from authorities to the public. Instead, there should be a focus on establishing robust, two-way communication channels that recognise the agency and localised knowledge of communities in shaping their own health outcomes. Such a shift can lead to more responsive, culturally sensitive and effective communication strategie. Seeking to enhance the synergy between community resilience and health emergency communication has the potential to foster greater trust, collaboration, resource accessibility and distribution, tailored communication, inclusivity, equity, engagement and feedback, but this requires further research and policy development. The model developed here holds promise in facilitating the coordination of grassroots community resilience efforts and promoting more efficient and adaptable health emergency communications tailored to different types of communities and population groups. The subsequent phase of this study involves roundtable discussions with community organisations, health communicators and policymakers (in May and November 2024). These collaborative endeavours aim to build a community of practice for further research, knowledge exchange and innovation.

## Supplementary Information

Additional file 1. PRISMA 2020 checklist.

Additional file 2. Key search terms.

Additional file 3. Bibliography (summary tables of all included articles).

## Data Availability

All data generated or analysed during this study are included in this published article and its supplementary information files.

## Abbreviations

AHA: American Hospital Association
CDCP: Centre for Disease Control and Prevention
DRR: Disaster Risk Reduction
ICT: Information Communication Technology
IMS: Incident Management System
LRF: Local Resilience Forum
NHS: National Health Service
PAR: Participatory Action Research
RCCE: Risk Communication and Community Engagement
SDG: Sustainable Development Goals
SES: Socioeconomic Status
UK: United Kingdom
UN: United Nations
US: United States of America
WHO: World Health Organization
