# Supplementary material for: Strengthening the relationship between community resilience and health emergency communication: a systematic review
Source: BMC Glob Public Health. 2024 Nov 27;2:79. doi: 10.1186/s44263-024-00112-y (PMC11622909; doi:10.1186/s44263-024-00112-y)
Supplement: Supplementary file 2 — Additional file 2. Key search terms. [file 44263_2024_112_MOESM2_ESM.docx]

| **Key Search Terms** | | |
| --- | --- | --- |
| Health emergency communication  Emergency health communication  Crisis communication  Public health messaging  Emergency information dissemination  Health promotion during emergencies  Disease prevention communication  Health behaviour change communication  Emergency preparedness communication  Disaster preparedness communication  Emergency response communication | AND | Community resilience  Community mobilization/mobilisation  Community efforts for resilience  Community collaboration for resilience  Self-support within communities  Social infrastructure resilience  Building community resilience  Strengthening community resilience  Promoting community resilience  Enhancing community resilience  Fostering community resilience |
| **Search string:**  ("Health emergency communication" OR "Emergency health communication" OR "Crisis communication" OR "Public health messaging" OR "Emergency information dissemination" OR "Health promotion during emergencies" OR "Disease prevention communication" OR "Health behaviour change communication" OR "Emergency preparedness communication" OR "Disaster preparedness communication" OR "Emergency response communication")  AND  ("Community resilience" OR "Community mobilization" OR “Community mobilisation” OR "Community efforts for resilience" OR "Community collaboration for resilience" OR "Self-support within communities" OR "Social infrastructure resilience" OR "Building community resilience" OR "Strengthening community resilience" OR "Promoting community resilience" OR "Enhancing community resilience" OR "Fostering community resilience") | | |
