# Supplementary material for: Strengthening the relationship between community resilience and health emergency communication: a systematic review
Source: BMC Glob Public Health. 2024 Nov 27;2:79. doi: 10.1186/s44263-024-00112-y (PMC11622909; doi:10.1186/s44263-024-00112-y)
Supplement: Supplementary file 3 — Additional file 3. Bibliography (summary tables of all included articles). [file 44263_2024_112_MOESM3_ESM.docx]

**Additional file 3. Bibliography of included articles**

**THEMES**

Theme 1: The relationship between emergency communication and community resilience

- Building trust and collaboration within communities (22 references)
- Identifying resources and their distribution (14 references)
- Tailoring communication strategies (12 references)
- Considering inclusion and equity (8 references)
- Community engagement and feedback (8 references)

Theme 2: Strategies and interventions to enhance community resilience in health emergency communication

- Facilitating community structures as channels for communication (9 references)
- Respecting personal and private boundaries in health communication (6 references)
- Targeting outreach for effective crisis communication (4 references)
- Building resilience through training and communication initiatives (3 references)
- Demonstrating commitment to equity and inclusion in communication (3 references)

**GRADE Criteria**

Categories A-D: High-, Moderate-, Low-, Very low-

A Systematic review of randomized controlled trials

Individual randomized controlled trials

B Systematic review of cohort studies of ‘exposed’ and ‘unexposed’ subjects

Individual cohort study/low quality randomized control studies

Systematic review of case-control studies

Individual case-control studies

C Case series, low-quality cohort or case-control studies

D Expert opinions based on non-systematic reviews of results or studies

Judgements about each of the quality of evidence factors assessed were (graded up or down):

🡫 Risk of bias (limitations in study design or execution)

🡫 Inconsistency of results (e.g., inconclusive or conflicting)

🡫 Indirectness of evidence (to the research question/topic)

🡫 Imprecision of results

🡫 Publication bias (e.g., not peer reviewed)

🡩 Factors that increase the quality of evidence e.g. multiple sources or large magnitude

GRADE Working Group. (2019). GRADE handbook for grading quality of evidence and strength of recommendations. Retrieved from <https://gdt.gradepro.org/app/handbook/handbook.html>

**Theme 1: The relationship between emergency communication and community resilience**

**Building trust and collaboration within communities**

| **Reference** | **Abstract** | **GRADE** |
| --- | --- | --- |
| Links JM, Schwartz BS, Lin S, Kanarek N, Mitrani-Reiser J, Sell TK, Watson CR, Ward D, Slemp C, Burhans R, Gill K. COPEWELL: a conceptual framework and system dynamics model for predicting community functioning and resilience after disasters. Disaster medicine and public health preparedness. 2018 Feb;12(1):127-37. | Objective: Policy-makers and practitioners have a need to assess community resilience in disasters. Prior efforts conflated resilience with community functioning, combined resistance and recovery (the components of resilience) and relied on a static model for what is inherently a dynamic process. We sought to develop linked conceptual and computational models of community functioning and resilience after a disaster. Methods: We developed a system dynamics computational model that predicts community functioning after a disaster. The computational model outputted the time course of community functioning before, during, and after a disaster, which was used to calculate resistance, recovery, and resilience for all US counties. Results: The conceptual model explicitly separated resilience from community functioning and identified all key components for each, which were translated into a system dynamics computational model with connections and feedbacks. The components were represented by publicly available measures at the county level. Baseline community functioning, resistance, recovery, and resilience evidenced a range of values and geographic clustering, consistent with hypotheses based on the disaster literature. Conclusions: The work is transparent, motivates ongoing refinements, and identifies areas for improved measurements. After validation, such a model can be used to identify effective investments to enhance community resilience. (Disaster Med Public Health Preparedness. 2018;12:127–137) | Grade C |
| Berkes F, Folke C, Colding J, editors. Linking social and ecological systems: management practices and social mechanisms for building resilience. Cambridge University Press; 2000 Apr 13. | It is usually the case that scientists examine either ecological systems or social systems, yet the need for an interdisciplinary approach to the problems of environmental management and sustainable development is becoming increasingly obvious. Developed under the auspices of the Beijer Institute in Stockholm, this new book analyses social and ecological linkages in selected ecosystems using an international and interdisciplinary case study approach. The chapters provide detailed information on a variety of management practices for dealing with environmental change. Taken as a whole, the book will contribute to the greater understanding of essential social responses to changes in ecosystems, including the generation, accumulation and transmission of ecological knowledge, structure and dynamics of institutions, and the cultural values underlying these responses. A set of new (or rediscovered) principles for sustainable ecosystem management is also presented. Linking Social and Ecological Systems will be of value to natural and social scientists interested in sustainability. | Grade D |
| Boin A, Lodge M. Designing resilient institutions for transboundary crisis management: A time for public administration. Public administration. 2016 Jun;94(2):289-98. | In the wake of severe climatic events and terrorist acts, and the emergence of dangerous technologies, communities, nations, and global organizations have diligently sought to create strategies to prepare for such events. Designing Resilience presents case studies of extreme events and analyzes the ability of affected individuals, institutions, governments, and technological systems to cope with disaster. This volume defines resilience as it relates to disaster management at specific stages: mitigation, prevention, preparation, and response and recovery. The book illustrates models by which to evaluate resilience at levels ranging from individuals to NGOs to governmental jurisdictions and examines how resilience can be developed and sustained. A group or nation's ability to withstand events and emerge from them with their central institutions intact is at the core of resilience. Quality of response, capacity to improvise, coordination, flexibility, and endurance are also determinants. Individual case studies, including Hurricane Katrina in the United States, the London bombings, and French preparedness for the Avian flu, demonstrate effective and ineffective strategies. The contributors reveal how the complexity and global interconnectivity of modern systems-whether they are governments, mobile populations, power grids, financial systems, or the Internet-have transcended borders and created a new level of exposure that has made them especially vulnerable to extreme events. Yet these far-reaching global systems also possess the ability to alert and respond at greater speeds than ever before. The authors analyze specific characteristics of resilient systems-the qualities they possess and how they become resilient-to determine if there are ways to build a system of resilience from the ground up. As such, Designing Resilience will inform a broad range of students and scholars in areas of public administration, public policy, and the social sciences. | Grade C |
| Bzdok D, Dunbar RI. The neurobiology of social distance. Trends in cognitive sciences. 2020 Sep 1;24(9):717-33. | Never before have we experienced social isolation on such a massive scale as we have in response to coronavirus disease 2019 (COVID-19). However, we know that the social environment has a dramatic impact on our sense of life satisfaction and well-being. In times of distress, crisis, or disaster, human resilience depends on the richness and strength of social connections, as well as on active engagement in groups and communities. Over recent years, evidence emerging from various disciplines has made it abundantly clear: perceived social isolation (i.e., loneliness) may be the most potent threat to survival and longevity. We highlight the benefits of social bonds, the choreographies of bond creation and maintenance, as well as the neurocognitive basis of social isolation and its deep consequences for mental and physical health. | Grade D |
| Comfort LK. Crisis management in hindsight: Cognition, communication, coordination, and control. Public administration review. 2007 Dec;67:189-97. | This article argues that cognition is central to performance in emergency management. Cognition is defined as the capacity to recognize the degree of emerging risk to which a community is exposed and to act on that information. Using the case of Hurricane Katrina to illustrate the collapse of the standard model of emergency management without a clear focus on the role of cognition, the author reframes the concept of intergovernmental crisis management as a complex, adaptive system. That is, the system needs to adjust and adapt its performance to fit the demands of an ever-changing physical, engineered, and social environment. The terms of cognition, communication, coordination, and control are redefined in ways that fit the reality of practice in extreme events. A reframed intergovernmental crisis management system may be conceived as a dynamic interorganizational system that is characterized by four primary decision points: (1) detection of risk, (2) recognition and interpretation of risk for the immediate context, (3) communication of risk to multiple organizations in a wider region, and (4) self-organization and mobilization of a collective, community response system to reduce risk and respond to danger. | Grade D |
| Corbin, J. H., Oyene, U. E., Manoncourt, E., Onya, H., Kwamboka, M., Amuyunzu-Nyamongo, M., Sørensen, K., Mweemba, O., Barry, M. M., Munodawafa, D., Bayugo, Y. V., Huda, Q., Moran, T., Omoleke, S. A., Spencer-Walters, D., & Van den Broucke, S. (2021). A health promotion approach to emergency management: effective community engagement strategies from five cases. Health promotion international, 36(Supplement_1), i24–i38. | Community engagement is crucial for controlling disease outbreak and mitigating natural and industrial disasters. The COVID-19 pandemic has reconfirmed the need to elevate community engagement to build equity, trust and sustained action in future health promotion preparedness strategies. Using the health promotion strategy of strengthening community action enhances the opportunity for better outcomes. There is, therefore, a need to improve our understanding of community engagement practices during crises, scale-up good community engagement initiatives, and improve and sustain people-centered approaches to emergency responses. This paper presents five case studies from the United States, Singapore, Sierra Leone, Kenya and South Africa that demonstrate the potential strengths that can be nurtured to build resilience in local communities to help mitigate the impact of disasters and emergencies. The case studies highlight the importance of co-developing relevant education and communication strategies, amplifying the role of community leaders, empowering community members to achieve shared goals, assessing and adapting to changing contexts, pre-planning and readiness for future emergencies and acknowledgement of historic context. | Grade D |
| Cunsolo Willox, A., Harper, S. L., Ford, J. D., Landman, K., Houle, K., & Edge, V. L. (2012). "From this place and of this place:” Climate change, sense of place, and health in Nunatsiavut, Canada. Social Science & Medicine, 75(3), 538–547. | As climate change impacts are felt around the globe, people are increasingly exposed to changes in weather patterns, wildlife and vegetation, and water and food quality, access and availability in their local regions. These changes can impact human health and well-being in a variety of ways: increased risk of foodborne and waterborne diseases; increased frequency and distribution of vector-borne disease; increased mortality and injury due to extreme weather events and heat waves; increased respiratory and cardiovascular disease due to changes in air quality and increased allergens in the air; and increased susceptibility to mental and emotional health challenges. While climate change is a global phenomenon, the impacts are experienced most acutely in place; as such, a sense of place, place-attachment, and place-based identities are important indicators for climate-related health and adaptation. Representing one of the first qualitative case studies to examine the connections among climate change, a changing sense of place, and health in an Inuit context, this research draws data from a multi-year community-driven case study situated in the Inuit community of Rigolet, Nunatsiavut, Canada. Data informing this paper were drawn from the narrative analysis of 72 in-depth interviews conducted from November 2009 to October 2010, as well as from the descriptive analysis of 112 questionnaires from a survey in October 2010 (95% response rate). The findings illustrated that climate change is negatively affecting feelings of place attachment by disrupting hunting, fishing, foraging, trapping, and traveling, and changing local landscapes—changes which subsequently impact physical, mental, and emotional health and well-being. These results also highlight the need to develop context-specific climate-health planning and adaptation programs, and call for an understanding of place-attachment as a vital indicator of health and well-being and for climate change to be framed as an important determinant of health. | Grade C |
| Imesha Dharmasena MG, Toledano M, Weaver CK. The role of public relations in building community resilience to natural disasters: perspectives from Sri Lanka and New Zealand. Journal of Communication Management. 2020 Oct 23;24(4):301-17. | Purpose: The paper identifies a role for public relations in disaster management by analysing disaster and communication managers' understanding of community resilience and their use of communication in the context of two different cultural environments. Design/methodology/approach: The research study comprised 51 in-depth qualitative interviews with disaster managers in Sri Lanka and New Zealand, which were thematically analysed using the software programme NVivo 10. Findings: The study identified cultural differences in Sri Lanka and New Zealand that impact on how managers' communicate in natural disaster situations. The findings indicated that public relations’ understanding of communities’ cultures, their communication, networking and lobbying skills could further enhance the effectiveness of efforts to build community resilience to disasters. Research limitations/implications: Nations are complex multicultural realities; the findings cannot be generalized to make claims about how natural disasters are managed in different national contexts. Practical implications: The paper identifies the unrealized potential of public relations’ expertise in communication, community relations, networking and lobbying to contribute to building community resilience to natural disasters. Social implications: By supporting efforts to build community resilience to disasters, public relations practitioners can contribute to social well-being in times of catastrophic natural disasters. Originality/value: The paper adds an innovative perspective to public relations crisis literature by identifying the potential contribution of public relations’ concepts and practices to build community resilience to natural disasters. It demonstrates how sociocultural differences may affect disaster communication strategies. | Grade C |
| Drury J, Carter H, Cocking C, Ntontis E, Tekin Guven S, Amlôt R. Facilitating collective psychosocial resilience in the public in emergencies: Twelve recommendations based on the social identity approach. Frontiers in public health. 2019 Jun 4;7:141. | Disaster myths are said to be widespread and consequential. However, there has been little research on whether those involved in public safety and emergency response believe them. A survey examined how far police officers, civilian safety professionals, sports event stewards and comparison samples from the public believe the myths “mass panic,” “civil disorder,” and “helplessness.” Respondents endorsed the first two myths. However, they rejected the myth of helplessness and endorsed the view that emergency crowds display resilience. Despite these contradictions in stated beliefs, there was also evidence of ideological coherence: each model of mass emergency behavior (maladaptive vs. resilient) was linked to a model of crowd management (coercive and paternalistic vs. mass-democratic). The practical implications of these findings are discussed. | Grade C |
| Dulebohn JH, Bommer WH, Liden RC, Brouer RL, Ferris GR. A meta-analysis of antecedents and consequences of leader-member exchange: Integrating the past with an eye toward the future. Journal of management. 2012 Nov;38(6):1715-59. | Although leader-member exchange (LMX) was identified in the literature nearly 40 years ago, a comprehensive empirical examination of its antecedents and consequences has not been conducted. The authors’ examination included 247 studies, containing 290 samples, and 21 antecedents and 16 consequences of LMX quality. Results indicated that while leader behaviors and perceptions, follower characteristics, interpersonal relationship characteristics, and contextual variables represent significant groups of LMX antecedents, leader variables explained the most variance in LMX quality. Moderator analyses revealed that the particular LMX scale, country of participants, and work setting studied did not produce meaningful influences on the relationships in the meta-analysis. However, power distance and individualism did moderate some of these relationships. To provide continuity with the LMX meta-analyses and conceptual reviews that have focused on LMX consequences, the authors tested a number of mediation models. The results demonstrated that LMX frequently plays a mediating role in the relationships where mediation could be tested. (PsycINFO Database Record (c) 2016 APA, all rights reserved) | Grade C |
| Folke C. Resilience: The emergence of a perspective for social–ecological systems analyses. Global environmental change. 2006 Aug 1;16(3):253-67. | The resilience perspective is increasingly used as an approach for understanding the dynamics of social–ecological systems. This article presents the origin of the resilience perspective and provides an overview of its development to date. With roots in one branch of ecology and the discovery of multiple basins of attraction in ecosystems in the 1960–1970s, it inspired social and environmental scientists to challenge the dominant stable equilibrium view. The resilience approach emphasizes non-linear dynamics, thresholds, uncertainty and surprise, how periods of gradual change interplay with periods of rapid change and how such dynamics interact across temporal and spatial scales. The history was dominated by empirical observations of ecosystem dynamics interpreted in mathematical models, developing into the adaptive management approach for responding to ecosystem change. Serious attempts to integrate the social dimension is currently taking place in resilience work reflected in the large numbers of sciences involved in explorative studies and new discoveries of linked social–ecological systems. Recent advances include understanding of social processes like, social learning and social memory, mental models and knowledge–system integration, visioning and scenario building, leadership, agents and actor groups, social networks, institutional and organizational inertia and change, adaptive capacity, transformability and systems of adaptive governance that allow for management of essential ecosystem services. | Grade D |
| Gardiner SA, Martin P. Bringing organisations together during a pandemic: the case of an intersectoral community support group. Australian Health Review. 2021 Dec 10;46(1):121-5. | In early 2020, the COVID-19 pandemic emerged, posing multiple challenges to healthcare organisations and communities. The Darling Downs region in Queensland, Australia had its first positive case of COVID-19 confirmed in March 2020, which created understandable anxiety in the community. The Vulnerable Communities Group (VCG) was established to address this anxiety through open lines of communication to strengthen community resilience. This case study reports the evaluation of the VCG, plus lessons learned while establishing and running an intersectoral group, with stakeholders from more than 40 organisations, in response to the COVID-19 pandemic. An anonymous online survey with closed and open-ended questions was administered to participants. Data were subject to descriptive statistical tests and content analysis. Four categories were developed from the free text data for reporting: 'Knowledge is power', 'Beating isolation through partnerships and linkages', 'Sharing is caring', and 'Ripple effects'. Whilst open communication and collaboration are always essential, they can be critically important during times of crisis. The VCG initiative is one example of tackling the challenges of the COVID-19 pandemic through bringing the community together. What is known about the topic? The COVID-19 pandemic has caused immense strain on the global community. It has thrust healthcare professionals and community leaders into an unforeseen situation, with little information being available on how to effectively deal with the pandemic to negate its effects. What does this paper add? This paper reports on the establishment and evaluation of the Vulnerable Communities Group (VCG) in the Toowoomba, Darling Downs region of Queensland, Australia in response to the COVID-19 pandemic, using a community of practice framework. It provides insight into how to establish and retain an intersectoral community of practice group during rapidly changing and challenging times, such as during the COVID-19 pandemic. What are the implications for practitioners? Practitioners can use a community of practice framework to establish and evaluate an intersectoral group, as described in our paper, to enhance community connectedness to reduce isolation and share information and resources to help negate the challenges caused by the COVID-19 pandemic. | Grade C |
| Hall CE, Wehling H, Stansfield J, South J, Brooks SK, Greenberg N, Amlôt R, Weston D. Examining the role of community resilience and social capital on mental health in public health emergency and disaster response: a scoping review. BMC public health. 2023 Dec 12;23(1):2482. | The ability of the public to remain psychologically resilient in the face of public health emergencies and disasters (such as the COVID-19 pandemic) is a key factor in the effectiveness of a national response to such events. Community resilience and social capital are often perceived as beneficial and ensuring that a community is socially and psychologically resilient may aid emergency response and recovery. This review presents a synthesis of literature which answers the following research questions: How are community resilience and social capital quantified in research?; What is the impact of community resilience on mental wellbeing?; What is the impact of infectious disease outbreaks, disasters and emergencies on community resilience and social capital?; and, What types of interventions enhance community resilience and social capital? A scoping review procedure was followed. Searches were run across Medline, PsycInfo, and EMBASE, with search terms covering both community resilience and social capital, public health emergencies, and mental health. 26 papers met the inclusion criteria. The majority of retained papers originated in the USA, used a survey methodology to collect data, and involved a natural disaster. There was no common method for measuring community resilience or social capital. The association between community resilience and social capital with mental health was regarded as positive in most cases. However, we found that community resilience, and social capital, were initially negatively impacted by public health emergencies and enhanced by social group activities. Several key recommendations are proposed based on the outcomes from the review, which include: the need for a standardised and validated approach to measuring both community resilience and social capital; that there should be enhanced effort to improve preparedness to public health emergencies in communities by gauging current levels of community resilience and social capital; that community resilience and social capital should be bolstered if areas are at risk of disasters or public health emergencies; the need to ensure that suitable short-term support is provided to communities with high resilience in the immediate aftermath of a public health emergency or disaster; the importance of conducting robust evaluation of community resilience initiatives deployed during the COVID-19 pandemic. | Grade C |
| Jones J, Barry MM. Factors influencing trust and mistrust in health promotion partnerships. Global Health Promotion. 2018;25(2):16-24. | Partnerships between sectors can achieve better outcomes than can be achieved by individual partners working alone. Trust is necessary for partnerships to function effectively. Mistrust makes partnership working difficult, if not impossible. There has been little research into partnership functioning factors that influence trust and mistrust. This study aimed to identify these factors in health promotion partnerships. Data were collected from 337 partners in 40 health promotion partnerships using a postal survey. The questionnaire incorporated multi-dimensional scales designed to assess the contribution of factors that influence partnership trust and mistrust. Newly validated scales were developed for trust, mistrust and power. Multiple regression analysis was used to identify the significance of each factor to partnership trust and mistrust. Power was found to be the only predictor of partnership trust. Power, leadership, and efficiency were the most important factors influencing partnership mistrust. Power in partnerships must be shared or partners will not trust each other. Power-sharing and trust-building mechanisms need to be built into partnerships from the beginning and sustained throughout the collaborative process. | Grade C |
| Lim S, Nakazato H. The emergence of risk communication networks and the development of citizen health-related behaviors during the COVID-19 pandemic: Social selection and contagion processes. International Journal of Environmental Research and Public Health. 2020 Jun;17(11):4148. | Amid the novel coronavirus pandemic, a variety of public health strategies have been implemented by governments worldwide. However, the fact that strict government mandates focus on physical distancing does not mean that social connectedness for voluntary risk communication among citizens should be sacrificed. Furthermore, we lack an understanding of citizens' behaviors regarding the voluntary adoption of public health measures and the control of mental wellbeing in the age of physical distancing. Key variables in the response to the global pandemic are the emergence of risk deliberation networks, voluntary compliance with government guidelines, and the restoration of citizens' subjective health. However, little is known about how citizens' health-related behaviors coevolve with social connections for sharing information and discussing urgent pandemic issues. The findings show that selection and social influence mechanisms coexist by affecting each citizen's health-related behaviors and community-led risk discourses in the face of the urgent health crisis. | Grade D |
| Marfori MT, Campbell SL, Garvey K, McKeown S, Veitch M, Wheeler AJ, Borchers-Arriagada N, Johnston FH. Public health messaging during extreme smoke events: are we hitting the mark? Frontiers in Public Health. 2020 Sep 2;8:465. | Background: Emergency services working to protect communities from harm during wildfires aim to provide regular public advisories on the hazards from fire and smoke. However, there are few studies evaluating the success of public health communications regarding the management of smoke exposure. We explored the responses to smoke-related health advisories of people living in a severely smoke-affected region during extensive wildfires in Tasmania, Australia early in 2019. We also evaluated the acceptability of portable high efficiency particle air (HEPA) cleaners used in study participant's homes during the smoky period. Methods: We conducted semi-structured interviews with 24 households in the Huon Valley region of Tasmania following a severe smoke episode. These households were initially recruited into a HEPA cleaner study. Interviews were recorded, transcribed, and analyzed for common themes using an inductive framework approach. Results: Public health messaging during the 2019 wildfire event in Tasmania was widely shared and understood, with social media playing a central role. However, some participants expressed concerns about the timeliness and effectiveness of the recommended interventions, and some would have appreciated more detailed information about the health risks from smoke. Public messages and actions to protect households from wildfire threat were, at times, contradictory or dominated in coverage over the smoke messaging, and many participants were conflicted with the multiple public messages and action relating to the more serious perceived threat from the fire. Conclusions: Public messaging about smoke and health should continue to use multiple avenues of communication, with a focus on simple messages provided through social media. Messaging about the smoke hazard should be available from a trusted central source regarding all aspects of the wildfire emergency, with links to more detailed information including local air quality data alongside interpretation of the associated health risks. | Grade C |
| Miles L, Gordon R, Bang H. Blaming active volcanoes or active volcanic blame? Volcanic crisis communication and blame management in the Cameroon. Observing the Volcano World: Volcano Crisis Communication. 2018:395-409. | This chapter examines the key role of blame management and avoidance in crisis communication with particular reference to developing countries and areas that frequently experience volcanic episodes and disasters. In these contexts, the chapter explores a key paradox prevalent within crisis communication and blame management concepts that has been rarely tested in empirical terms (see De Vries 2004; Brändström 2016a). In particular, the chapter examines, what it calls, the ‘paradox of frequency’ where frequency of disasters leads to twin dispositions for crisis framed as either: (i) policy failure (active about volcanic blame on others), where issues of blame for internal incompetency takes centre stage, and blame management becomes a focus of disaster managers, and/or: (ii) as event failure (in this case, the blaming of lack of external capacity on active volcanoes and thereby the blame avoidance of disaster managers). Put simply, the authors investigate whether perceptions of frequency itself is a major determinant shaping the existence, operation, and even perceived success of crisis communication in developing regions, and countries experiencing regular disaster episodes. The authors argue frequency is important in shaping the behaviour of disaster managers and rather ironically as part of crisis communication can shape expectations of community resilience and (non)-compliance. In order to explore the implications of the ‘paradox of frequency’ further, the chapter examines the case of the Cameroon, where volcanic activity and events have been regular, paying particular attention to the major disasters in 1986 (Lake Nyos Disaster - LND) and 1999 (Mount Cameroon volcanic eruption - MCE). | Grade D |
| Norris, F. H., Stevens, S. P., Pfefferbaum, B., Wyche, K. F., & Pfefferbaum, R. L. (2008). Community resilience as a metaphor, theory, set of capacities, and strategy for disaster readiness. American Journal of Community Psychology, 41(1-2), 127-150. | Communities have the potential to function effectively and adapt successfully in the aftermath of disasters. Drawing upon literatures in several disciplines, we present a theory of resilience that encompasses contemporary understandings of stress, adaptation, wellness, and resource dynamics. Community resilience is a process linking a network of adaptive capacities (resources with dynamic attributes) to adaptation after a disturbance or adversity. Community adaptation is manifest in population wellness, defined as high and non-disparate levels of mental and behavioral health, functioning, and quality of life. Community resilience emerges from four primary sets of adaptive capacities--Economic Development, Social Capital, Information and Communication, and Community Competence--that together provide a strategy for disaster readiness. To build collective resilience, communities must reduce risk and resource inequities, engage local people in mitigation, create organizational linkages, boost and protect social supports, and plan for not having a plan, which requires flexibility, decision-making skills, and trusted sources of information that function in the face of unknowns. | Grade C |
| O'Sullivan TL, Kuziemsky CE, Corneil W, Lemyre L, Franco Z. The EnRiCH community resilience framework for high-risk populations. PLoS currents. 2014 Oct 2;6. | Introduction: Resilience has been described in many ways and is inherently complex. In essence, it refers to the capacity to face and do well when adversity is encountered. There is a need for empirical research on community level initiatives designed to enhance resilience for high-risk groups as part of an upstream approach to disaster management. In this study, we address this issue, presenting the EnRiCH Community Resilience Framework for High-Risk Populations. Methods: The framework presented in this paper is empirically-based, using qualitative data from focus groups conducted as part of an asset-mapping intervention in five communities in Canada, and builds on extant literature in the fields of disaster and emergency management, health promotion, and community development. Results: Adaptive capacity is placed at the centre of the framework as a focal point, surrounded by four strategic areas for intervention (awareness/communication, asset/resource management, upstream-oriented leadership, and connectedness/engagement). Three drivers of adaptive capacity (empowerment, innovation, and collaboration) cross-cut the strategic areas and represent levers for action which can influence systems, people and institutions through expansion of asset literacy. Each component of the framework is embedded within the complexity and culture of a community. Discussion: We present recommendations for how this framework can be used to guide the design of future resilience-oriented initiatives with particular emphasis on inclusive engagement across a range of functional capabilities. | Grade C |
| Paton D, Johnston D. Disaster resilience: an integrated approach. Charles C Thomas Publisher; 2017 Jun 12. | With regard to their utility in predicting the adoption of household hazard preparations, traditional approaches to public education directed at increasing awareness and/or risk perception have proven ineffective. Discusses reasons why this may have occurred from public education, vulnerability analysis, and community resilience perspectives and outlines strategies for enhancing preparedness. Describes a model of resilience to hazard effects that has been tested in different communities and for different hazards (toxic waste, environmental degradation and volcanic hazards). Drawing upon the health education literature, introduces a model for promoting the adoption on preparatory behaviour. Discusses links between these models, and the need for their implementation within a community development framework. | Grade D |
| Villalonga-Olives E, Kawachi I. The dark side of social capital: A systematic review of the negative health effects of social capital. Social science & medicine. 2017 Dec 1;194:105-27. | There is a growing literature demonstrating the health benefits of social capital (defined as the resources accessed through social connections). However, social capital is also acknowledged to be a "double-edged" phenomenon, whose effects on health are not always positive. We sought to systematically review studies that have found a negative (i.e. harmful) association between social capital and health outcomes. Our objective was to classify the different types of negative effects, following a framework originally proposed by Portes (1998). We conducted a literature search in PubMed, Embase and PsychInfo. We identified 3530 manuscripts. After detailed review, we included 44 articles in our systematic review. There are at least two negative consequences of social capital besides the classification proposed by Portes: behavioral contagion and cross-level interactions between social cohesion and individual characteristics. When leveraging the concept of social capital for health promotion interventions, researchers need to take account of these potential "downsides" for health outcomes. | Grade B |
| Wilson S, Fernandes-Jesus M, Young J, Drury J, Harris C, Graber R, Northorp S, O’Dwyer E, Walker C. A Social Capital Approach to Understanding Community Resilience during the Covid-19 Pandemic. InForum Community Psychology 2023 May 15. Forum Community Psychology. | As the world begins to take stock of the impact of Covid-19, this paper provides a critical review of the role of mutual aid groups in the UK community response. Drawing on interviews with community members and selective case studies, this position paper considers what forms of social capital impact on availability of mutual aid support in the community response to Covid-19. Based on our own experiences and Covid-19 specific research, we found that communities with social capital have been among the most organised in providing formal mutual aid, and sometimes this has extended to supporting the more marginalised and disadvantaged communities. The phenomenon of collective resilience in the pandemic, and in particular the activities of mutual aid groups as described in this paper, testify to the relevance of key concepts in social and community psychology. Without psychological ‘groupness’ there would be no adaptive community response. Further research is needed to better understand the role that social capital played in both the individual and community resilience of those offering and receiving mutual aid. This is of particular importance, given that communities lacking social capital are more vulnerable to social exclusion, in a global context where societal inequalities are widening. Whilst appreciating the limitations of social capital (particularly that it does not explain the new group relationships and forms of solidarity that have emerged), we argue that developing social capital, particularly bridging and linking social capital, can help to build community resilience and promote inclusions in communities bearing the collective economic and societal burden of the pandemic. | Grade B |

**Identifying resources and their distribution**

| **Reference** | **Abstract** | **GRADE** |
| --- | --- | --- |
| Adger WN, Hughes TP, Folke C, Carpenter SR, Rockstrom J. Social-ecological resilience to coastal disasters. Science. 2005 Aug 12;309(5737):1036-9. | Social and ecological vulnerability to disasters and outcomes of any particular extreme event are influenced by buildup or erosion of resilience both before and after disasters occur. Resilient social-ecological systems incorporate diverse mechanisms for living with, and learning from, change and unexpected shocks. Disaster management requires multilevel governance systems that can enhance the capacity to cope with uncertainty and surprise by mobilizing diverse sources of resilience. | Grade D |
| Adger WN. Social and ecological resilience: are they related? Progress in human geography. 2000 Sep;24(3):347-64. | This article defines social resilience as the ability of groups or communities to cope with external stresses and disturbances as a result of social, political and environmental change. This definition highlights social resilience in relation to the concept of ecological resilience which is a characteristic of ecosystems to maintain themselves in the face of disturbance. There is a clear link between social and ecological resilience, particularly for social groups or communities that are dependent on ecological and environmental resources for their livelihoods. But it is not clear whether resilient ecosystems enable resilient communities in such situations. This article examines whether resilience is a useful characteristic for describing the social and economic situation of social groups and explores potential links between social resilience and ecological resilience. The origins of this interdisciplinary study in human ecology, ecological economics and rural sociology are reviewed, and a study of the impacts of ecological change on a resource-dependent community in contemporary coastal Vietnam in terms of the resilience of its institutions is outlined. | Grade D |
| Aldrich DP, Meyer MA. Social capital and community resilience. American behavioral scientist. 2015 Feb;59(2):254-69. | Despite the ubiquity of disaster and the increasing toll in human lives and financial costs, much research and policy remain focused on physical infrastructure–centered approaches to such events. Governmental organizations such as the Department of Homeland Security, United States Federal Emergency Management Agency, United States Agency for International Development, and United Kingdom’s Department for International Development continue to spend heavily on hardening levees, raising existing homes, and repairing damaged facilities despite evidence that social, not physical, infrastructure drives resilience. This article highlights the critical role of social capital and networks in disaster survival and recovery and lays out recent literature and evidence on the topic. We look at definitions of social capital, measurement and proxies, types of social capital, and mechanisms and application. The article concludes with concrete policy recommendations for disaster managers, government decision makers, and nongovernmental organizations for increasing resilience to catastrophe through strengthening social infrastructure at the community level. | Grade C |
| Bruneau M, Chang SE, Eguchi RT, Lee GC, O'Rourke TD, Reinhorn AM, Shinozuka M, Tierney K, Wallace WA, Von Winterfeldt D. A framework to quantitatively assess and enhance the seismic resilience of communities. Earthquake spectra. 2003 Nov;19(4):733-52. | This paper presents a conceptual framework to define seismic resilience of communities and quantitative measures of resilience that can be useful for a coordinated research effort focusing on enhancing this resilience. This framework relies on the complementary measures of resilience: “Reduced failure probabilities,” “Reduced consequences from failures,” and “Reduced time to recovery.” The framework also includes quantitative measures of the “ends” of robustness and rapidity, and the “means” of resourcefulness and redundancy, and integrates those measures into the four dimensions of community resilience—technical, organizational, social, and economic—all of which can be used to quantify measures of resilience for various types of physical and organizational systems. Systems diagrams then establish the tasks required to achieve these objectives. This framework can be useful in future research to determine the resiliency of different units of analysis and systems, and to develop resiliency targets and detailed analytical procedures to generate these values. | Grade D |
| Cutter SL, Barnes L, Berry M, Burton C, Evans E, Tate E, Webb J. A place-based model for understanding community resilience to natural disasters. Global environmental change. 2008 Oct 1;18(4):598-606. | There is considerable research interest on the meaning and measurement of resilience from a variety of research perspectives including those from the hazards/disasters and global change communities. The identification of standards and metrics for measuring disaster resilience is one of the challenges faced by local, state, and federal agencies, especially in the United States. This paper provides a new framework, the disaster resilience of place (DROP) model, designed to improve comparative assessments of disaster resilience at the local or community level. A candidate set of variables for implementing the model are also presented as a first step towards its implementation. | Grade D |
| Cutter SL, Boruff BJ, Shirley WL. Social vulnerability to environmental hazards. In Hazards vulnerability and environmental justice 2012 May 4 (pp. 143-160). Routledge. | Objective. County-level socioeconomic and demographic data were used to construct an index of social vulnerability to environmental hazards, called the Social Vulnerability Index (SoVI) for the United States based on 1990 data. Methods. Using a factor analytic approach, 42 variables were reduced to 11 independent factors that accounted for about 76 percent of the variance. These factors were placed in an additive model to compute a summary score—the Social Vulnerability Index. Results. There are some distinct spatial patterns in the SoVI, with the most vulnerable counties clustered in metropolitan counties in the east, south Texas, and the Mississippi Delta region. Conclusion. Those factors that contribute to the overall score often are different for each county, underscoring the interactive nature of social vulnerability—some components increase vulnerability; others moderate the effects. | Grade C |
| Kar B, Cochran DM, editors. Risk communication and community resilience. Routledge; Abingdon, Oxon. 2019 May 28. | Risk communication is the exchange of information among stakeholders about an impending disaster and its risks to help individuals take appropriate actions to mitigate hazard impacts. While traditional risk communication follows a command and control structure such that information from hierarchical and vertically integrated organizations is disseminated to broader community, social media uses a decentralized, collaborative, and network based communication approach. The growth of information and communication technologies has made social media a popular channel for disseminating alert and warning messages both by citizens and agencies. However, social media suffers from spreading rumors and hoaxes. To minimize rumors and increase citizen communication, a science gateway (Cyber-Infrastructure for GeoInformatics and Community Resilience) has been deployed. This gateway, resulted from research conducted along the Mississippi Gulf Coast communities, incorporates citizen science to evaluate warning message sources, message contents and dissemination channels to increase public response to warnings. This gateway built on the social construct of risk communication provides opportunities to citizens to share data and information about a hazard and participate in building community resilience. Copyright © 2015 John Wiley & Sons, Ltd. | Grade C |
| N. Lam NS, Reams M, Li K, Li C, Mata LP. Measuring community resilience to coastal hazards along the Northern Gulf of Mexico. Natural hazards review. 2016 Feb 1;17(1):04015013. | The abundant research examining aspects of social-ecological resilience, vulnerability, and hazards and risk assessment has yielded insights into these concepts and suggested the importance of quantifying them. Quantifying resilience is complicated by several factors including the varying definitions of the term applied in the research, difficulties involved in selecting and aggregating indicators of resilience, and the lack of empirical validation for the indices derived. This paper applies a new model, called the resilience inference measurement (RIM) model, to quantify resilience to climate-related hazards for 52 U.S. counties along the northern Gulf of Mexico. The RIM model uses three elements (exposure, damage, and recovery indicators) to denote two relationships (vulnerability and adaptability) and employs both K-means clustering and discriminant analysis to derive the resilience rankings, thus enabling validation and inference. The results yielded a classification accuracy of 94.2% with 28 predictor variables. The approach is theoretically sound and can be applied to derive resilience indices for other study areas at different spatial and temporal scales. | Grade C |
| Organisation for Economic Co-operation and Development (OECD) Overview Paper on Resilient Communities and Societies, 2014. https://www.oecd.org/mcm/C-MIN(2014)7-ENG.pdf | The 2008 economic and financial crisis highlighted the importance of strengthening the resilience of our economies, societies and institutions. Resilience is a broad concept, centred on the ability not only to resist and recover from adverse shocks, but also to “bounce back” stronger than before, and to learn from the experience. Resilience is also multidimensional, encompassing a range of interconnected factors and conditions. Strengthening resilience is all the more essential today in the face of increasing policy complexity and interconnectedness, deep-seated demographic and technological trends, and growing environmental pressures, all of which increase the likelihood of some critical event having negative impacts on economic growth and well-being. Being resilient involves understanding the sources of risks and opportunities and learning to cope with uncertainty. There is no single methodological approach for measuring resilience and concrete analysis and recommendations are best developed within specific policy areas. This paper addresses resilience across four broad dimensions covering economic, social, institutional and environmental issues. Each of these dimensions is relevant in its own way and they are all strongly interconnected, reflecting the capacity of individuals, organisations and systems to withstand and recover from shocks. | Grade B |
| Page MJ, McKenzie JE, Bossuyt PM, Boutron I, Hoffmann TC, Mulrow CD, Shamseer L, Tetzlaff JM, Akl EA, Brennan SE, Chou R. The PRISMA 2020 statement: an updated guideline for reporting systematic reviews. BMJ. 2021 Mar 29;372. doi: https://doi.org/10.1136/bmj.n71 | Primary education in the United States is an important cornerstone for communities, families, and students. For this reason, the relationships that are fostered through primary education facilities can be used to attend to and mitigate disasters. Social media innovations make these relationships even more important by allowing for near-instant communication among schools and community members. Yet very little literature has examined the unique communication environment that schools face or how they prepare to address crises. This chapter examines risk and crisis communication in K-12 education through an in-depth analysis of schools in a large East Coast state in the United States. The evaluation reports on the content analysis of 611 crisis-related social media posts from 22 school systems and on a survey of 132 teachers, school nurses, principals, superintendents, and school safety administrators about their experiences and communication preparation for crises. Findings provide insights into how schools can improve risk and crisis communication preparation. Such efforts not only would contribute to keeping children safe but would do the same for nearby communities that often rely on schools as centralized locations for post-disaster assistance. | Grade C |
| Paton D, Johnston D. Disaster resilience: an integrated approach. Charles C Thomas Publisher; Springfield, Illinois. 2017 Jun 12. | Events such as the 2004 Indian Ocean Tsunami, Hurricane Katrina in 2005 and the Japanese earthquakes and tsunamis in 2011 have provided unfortunate reminders of the susceptibility of many communities to devastating losses from natural hazards. These events provided graphic illustrations of how extreme hazard events adversely impact on people, affect communities and disrupt the community and societal mechanisms that serve to organize and sustain community capacities and functions. However, there is much that communities can do to mitigate their risk and manage disaster consequences. The construct that epitomizes how this is done is resilience. The contents of this volume provide valuable insights into how societal resilience can be developed and sustained. This considerably expanded new edition presents major topics of: Coexisting with Natural Hazards; Urban Resilience in Asia; Lifelines and Urban Resilience; Business Continuity in Disaster; Hazard Mitigation in Communities; Hazard Readiness and Resilience; Child Citizenship in Disaster Risk; Old Age and Resilience; Gender and Disaster Resilience; Impact of High Functionality on Resilience; Art and Resilience; Cross-Cultural Perspectives and Coping with Hazards; Religious Practices and Resilience; Living in Harmony with our Environment; Critical Incidence Response; Governance; Heat Wave Resilience; Wildfire Disaster Resilience; and Progress and Challenges to Disaster Risk Reduction and Resilience. This exceptional book brings together contributions from international experts in core areas and includes chapters that provide and overarching framework within which the need for interrelationships between levels to be developed is discussed. The book will be an outstanding resource for those researching or teaching courses in emergency management, disaster management, community development, environmental planning and urban development. In addition, it will serve law enforcement and emergency agencies, welfare agencies, and professionals in applied psychology. | Grade C |
| Sherrieb K, Norris FH, Galea S. Measuring capacities for community resilience. Social indicators research. 2010 Nov;99:227-47. | The purpose of this study was to measure the sets of adaptive capacities for Economic Development and Social Capital in the Norris et al. (2008) community resilience model with publicly accessible population indicators. Our approach involved five steps. First, we conducted a literature review on measurements of the capacities. Second, we created an exhaustive “wish list” of relevant measures that operationalized the concepts presented in the literature. Third, we identified data sources and searched for archival, population-level data that matched our indicators. Fourth, we systematically tested correlations of indicators within and across the theoretical elements and used this information to select a parsimonious group of indicators. Fifth, we combined the indicators into composites of Economic Development and Social Capital and an additive index of Community Resilience using Mississippi county data and validated these against a well-established index of social vulnerability and aggregated survey data on collective efficacy. We found that our measure of community resilience capacities correlated favorably and as expected when validated with the archival and survey data. This study provides the first step in identifying existing capacities that may predict a community’s ability to “bounce back” from disasters, thereby reducing post-trauma health and mental health problems. | Grade B |
| Silver A. The use of social media in crisis communication. In Risk communication and community resilience 2019 May 28 (pp. 267-282). Routledge. | The fields of risk and crisis communication have undergone a rapid transformation over the last 20 years. With the development of the Internet, Web 2.0, and mobile phone applications, the traditional “expert-to-receiver” model of communication has become largely outdated. Social media has allowed citizens to actively engage in and contribute to crisis dialogues, thus becoming influential content creators themselves. This chapter will explore the various strengths and weaknesses of social media as a crisis communications tool. Several case studies are used to exemplify the use of social media during extreme events, including Hurricane Harvey, Hurricane Sandy, the Boston Marathon bombing, and the Goderich tornado. Issues of trust, credibility, misinformation management, crowdsourcing, and public safety are also explored. The chapter concludes with a discussion about social media and community resilience. It is emphasized that social media can facilitate self-organization, information seeking, and sharing and act as a platform for the psychosocial healing of survivors. Accordingly, social media has the potential to positively influence community resilience before, during, and after crises. | Grade B |
| United Nations Common Guidance on Helping Build Resilient Societies, 2020. New York, UN. https://unsdg.un.org/resources/un-common-guidance-helping-build-resilient-societies | A tool for disaster resilience planning: United Nations Office for Disaster Risk Reduction with the support of European Commission, IBM, AECOM and other partners and cities participating in the Making Cities Resilient Campaign have updated. The Disaster Resilience Scorecard for Cities: The Scorecard provides a set of assessments that allow local governments to assess their disaster resilience, structuring around UNDRR’s Ten Essentials for Making Cities Resilient. It also helps to monitor and review progress and challenges in the implementation of the Sendai Framework for Disaster Risk Reduction: 2015-2030 and supports the baseline analysis for preparation of the disaster risk reduction and resilience strategies. It offers the potential for scoring at two levels: • Level 1: Preliminary level , responding to key Sendai Framework targets and indicators, and with some critical sub-questions. This approach is suggested for use in a 1 to 2 day city multi-stakeholder workshop. In total there are 47 questions indicators, each with a 0 – 3 score; • Level 2: Detailed assessment . This approach is a multi-stakeholder exercise that may take 1 –4 months and can be a basis for a detailed city resilience action plan. The detailed assessment includes 117 indicator criteria, each with a score of 0 – 5. Note that the criterion in the detailed assessment may serve as helpful discussion prompts for a preliminary level workshop. While the Scorecard can be used as a standalone tool, it does require you to consider your city’s hazards and risks. Specifically, the Scorecard prompts you to identify “most probable” and “most severe” risk scenarios for each of your identified city hazards, or for a potential multi-hazard event. | Grade B |

**Tailoring communication strategies**

| **Reference** | **Abstract** | **GRADE** |
| --- | --- | --- |
| Acosta JD, Burgette L, Chandra A, Eisenman DP, Gonzalez I, Varda D, Xenakis L. How community and public health partnerships contribute to disaster recovery and resilience. Disaster medicine and public health preparedness. 2018 Oct;12(5):635-43. | Objective: To summarize ways that networks of community-based organizations (CBO), in partnership with public health departments, contribute to community recovery from disaster. Methods: The study was conducted using an online survey administered one and 2 years after Hurricane Sandy to the partnership networks of 369 CBO and the New York Department of Health and Mental Hygiene. The survey assessed the structure and durability of networks, how they were influenced by storm damage, and whether more connected networks were associated with better recovery outcomes. Results: During response and recovery, CBOs provide an array of critical public health services often outside their usual scope. New CBO partnerships were formed to support recovery, particularly in severely impacted areas. CBOs that were more connected to other CBOs and were part of a long-term recovery committee reported greater impacts on the community; however, a partnership with the local health department was not associated with recovery impacts. Conclusion: CBO partners are flexible in their scope of services, and CBO partnerships often emerge in areas with the greatest storm damage, and subsequently the greatest community needs. National policies will advance if they account for the dynamic and emergent nature of these partnerships and their contributions and clarify the role of government partners. (Disaster Med Public Health Preparedness. 2018;12:635-643). | Grade D |
| Bukar UA, Jabar MA, Sidi F, Nor RB, Abdullah S, Ishak I. How social media crisis response and social interaction is helping people recover from Covid-19: an empirical investigation. Journal of computational social science. 2022 May:1-29. | Community resilience following a crisis has become essential to avoid panic. In contrast, social media usage has been practical to improve public resilience. However, the impacts of social media crisis response and social interaction have not been fully addressed. Therefore, this study aims to investigate the effects of social media crisis communication on public resilience. The study data were collected through an online medium, and the final responses consist of 393 observations, mainly of Malaysians who have experienced Covid-19 isolation, quarantine, or lockdown. The assessments of the reflective measurement models based on path analysis in PLS-SEM are reliable and valid. The Cronbach’s alpha, rho_A, composite reliability, and discriminant validity revealed acceptable values. PLS prediction algorithm was run to assess the model’s predictive power, and the findings show that the predictive relevance is satisfactory. Furthermore, the IPMA was applied to evaluate the model’s usefulness, which compares the level of the variables from the performance scale mean value against the importance level. The result shows that all the variables are useful and reveal good performance. Thus, crisis management and communication activities should pay more attention to these variables for effective social media crisis communication. Thus, the study offers theoretical and practical implications in the field of social media-based crisis communication and crisis informatics. | Grade C |
| Chandra A, Acosta J, Howard S, Uscher-Pines L, Williams M, Yeung D, Garnett J, Meredith LS. Building community resilience to disasters: A way forward to enhance national health security. Rand health quarterly. 2011;1(1). | Community resilience, or the sustained ability of a community to withstand and recover from adversity, has become a key policy issue at federal, state, and local levels, including in the National Health Security Strategy. Because resources are limited in the wake of an emergency, it is increasingly recognized that resilience is critical to a community's ability to reduce long recovery periods after an emergency. This article shares details of a report that provides a roadmap for federal, state, and local leaders who are developing plans to enhance community resilience for health security threats and describes options for building community resilience in key areas. Based on findings from a literature review and a series of community and regional focus groups, the authors provide a definition of community resilience in the context of national health security and a set of eight levers and five core components for building resilience. They then describe suggested activities that communities are pursuing and may want to strengthen for community resilience, and they identify challenges to implementation. | Grade B |
| Cutter SL, Barnes L, Berry M, Burton C, Evans E, Tate E, Webb J. A place-based model for understanding community resilience to natural disasters. Global environmental change. 2008 Oct 1;18(4):598-606. | There is considerable research interest on the meaning and measurement of resilience from a variety of research perspectives including those from the hazards/disasters and global change communities. The identification of standards and metrics for measuring disaster resilience is one of the challenges faced by local, state, and federal agencies, especially in the United States. This paper provides a new framework, the disaster resilience of place (DROP) model, designed to improve comparative assessments of disaster resilience at the local or community level. A candidate set of variables for implementing the model are also presented as a first step towards its implementation. | Grade C |
| Gaspar R, Domingos S, Brito D, Leiras G, Filipe J, Raposo B, Telo de Arriaga M. Striving for crisis resolution or crisis resilience? The crisis layers and thresholds model and Information and Communication Technology‐mediated social sensing for evidence‐based crisis management and communication. Human behavior and emerging technologies. 2021 Jan;3(1):40-52. | Do crisis evolve linearly through sequential one-directional stages that end with their resolution? Or are crisis, a set of nonlinear events with somewhat a chaotic nature, better represented as multilayer relapse cycles, that is, a series of dynamic processes and templates that evolve at different levels of analysis and can either go forward—achievement—or go back—relapses? Moreover, should crisis always move forward to reach their resolution or should we strive to achieve social systems resilience, grounded on learning and adaptation processes, that is, moving forward and backwards, until achieving it? To argument in favor of achieving crisis resilience, we propose a theoretical model—the crisis layers and thresholds (CLT) model grounded on the following assumptions: (a) individuals' evaluations and responses should be the basis/core of crisis management and crisis communication activities; (b) different concurrent psychosocial and organizational processes occur at different levels of analysis of a crisis, from a micro-individual level to a macro organization level; and (c) rather than striving for crisis resolution, we should strive for crisis resilience, preparing the social system for current and future emerging risks and crisis. To implement effective evidence-based crisis management and crisis communication in line with such assumptions, we also propose the CLT-ResiliScence approach, an Information and Communication Technology-mediated crisis sensing approach. This is based on monitoring “social sensors” data, particularly from social media, as an important source of information. Examples of this will be provided based on research on the current COVID-19 pandemic. | Grade D |
| Neelakantan V. Tamara Giles-Vernick and James LA Webb (eds), Global Health in Africa: Historical Perspectives on Disease Control, 2015: 220-221. | Global Health in Africa, the first exploratory study of global health initiatives in Africa, comprises contributions from scholars from a range of social and biomedical sciences. Unlike previous studies of African medical history, the contributions to this collection, which originated from a one-day workshop at Princeton in 2008, are neither restricted to a single disease, nor to a particular country. Nor do they advance a single argument. The editors define global health from the perspective of public health initiatives launched within Africa by outside actors such as colonial governments, international health organisations and foreign aid. The themes broached by the collection range from the history of health interventions in African nations with respect to epidemic diseases such as smallpox, cholera and HIV/AIDS, endemic diseases such as malaria and chronic non-infectious conditions particularly chronic malnutrition and injection drug use. The volume is thematically divided into three parts. The first three chapters in Part I of the collection critique the colonial-era public health interventions directed against smallpox, malaria and yaws. Jennifer Tappan's essay investigates how the tangled scientific understandings of severe childhood malnutrition between 1950 and 1974 led nutritionists to prescribe dried skim milk into the diets of malnourished infants, a suggestion which had disastrous health consequences. | Grade C |
| Lwin MO, Vijaykumar S, Fernando ON, Cheong SA, Rathnayake VS, Lim G, Theng YL, Chaudhuri S, Foo S. A 21st century approach to tackling dengue: Crowdsourced surveillance, predictive mapping and tailored communication. Acta tropica. 2014 Feb 1;130:100-7. | This paper describes a social media system to prevent dengue in Sri Lanka and potentially in the rest of the South and Southeast Asia regions. The system integrates three concepts of public health prevention that have thus far been implemented only in silos. First, the predictive surveillance component uses a computer simulation to forewarn health authorities and the general public about impending disease outbreaks. The civic engagement component allows the general public to use social media tools to interact and engage with health authorities by aiding them in surveillance efforts by reporting symptoms, mosquito bites and breeding sites using smartphone technologies. The health communication component utilizes citizen data gathered from the first two components to disseminate customized health awareness messages to enhance knowledge and increase preventive behaviors among citizens. The system, known as “Mo-Buzz,” will be made available on a host of digital platforms like simple mobile phones, smart phones and a website. We present challenges and lessons learnt including content validation, stakeholder collaborations and applied trans-disciplinary research. | Grade B |
| National Academies, Policy, Global Affairs, Committee on Science, Public Policy, Committee on Increasing National Resilience to Hazards, Disasters. Disaster resilience: A national imperative. National Academies Press; 2012 Dec 29. | No person or place is immune from disasters or disaster-related losses. Infectious disease outbreaks, acts of terrorism, social unrest, or financial disasters in addition to natural hazards can all lead to large-scale consequences for the nation and its communities. Communities and the nation thus face difficult fiscal, social, cultural, and environmental choices about the best ways to ensure basic security and quality of life against hazards, deliberate attacks, and disasters. Beyond the unquantifiable costs of injury and loss of life from disasters, statistics for 2011 alone indicate economic damages from natural disasters in the United States exceeded $55 billion, with 14 events costing more than a billion dollars in damages each. | Grade B |
| Norris, F. H., Stevens, S. P., Pfefferbaum, B., Wyche, K. F., & Pfefferbaum, R. L. (2008). Community resilience as a metaphor, theory, set of capacities, and strategy for disaster readiness. American Journal of Community Psychology, 41(1-2), 127-150. | Communities have the potential to function effectively and adapt successfully in the aftermath of disasters. Drawing upon literatures in several disciplines, we present a theory of resilience that encompasses contemporary understandings of stress, adaptation, wellness, and resource dynamics. Community resilience is a process linking a network of adaptive capacities (resources with dynamic attributes) to adaptation after a disturbance or adversity. Community adaptation is manifest in population wellness, defined as high and non-disparate levels of mental and behavioral health, functioning, and quality of life. Community resilience emerges from four primary sets of adaptive capacities--Economic Development, Social Capital, Information and Communication, and Community Competence--that together provide a strategy for disaster readiness. To build collective resilience, communities must reduce risk and resource inequities, engage local people in mitigation, create organizational linkages, boost and protect social supports, and plan for not having a plan, which requires flexibility, decision-making skills, and trusted sources of information that function in the face of unknowns. | Grade C |
| Olshansky RB. Planning after hurricane Katrina. Journal of the American Planning Association. 2006 Jun 30;72(2):147-53. | Hurricane Katrina was the greatest urban and regional disaster in U.S. history. The rebuilding of New Orleans and surrounding areas of Louisiana and Mississippi will require the largest and most complex planning effort in my lifetime. To succeed, we must learn from disasters of the past, while also applying the planning knowledge of the present. From past disasters, we know that successful reconstruction requires both outside funding and local citizen involvement. As planners, we know that the processes should be rich in data, imagination, communication, and participation. Optimistically, a new New Orleans will involve improved flood safety, revitalized neighborhoods, housing opportunities for all, and equitable treatment of all residents. Planners have an obligation to take an active role and advocate for the funding and full participation necessary to achieve these goals. The alternative would be a city that is poor, unsafe, and unequal. This is the greatest planning problem most of us have ever seen, and it warrants a correspondingly large response. | Grade B |
| Sharpe J. Learning to be practical: A guided learning approach to transform student community resilience when faced with natural hazard threats. Observing the Volcano World: Volcano Crisis Communication. 2018:715-31. | This chapter seeks to explore how creative use of educational resources can challenge students to take responsibility for their own preparedness and safety in response to natural hazard risks. A brief context for the need for learning-focused rather than education-focused curriculum is explored before the England and Wales context is brought into focus. Two methods for transforming learning around the theme of natural hazard risk and response are offered: A film project in which students produce films by and for children and youth and a ‘Go-Bag’ project in which students take on a practical task of making up a real emergency bag. By guiding student learning, but allowing it to develop inside a reasonable framework, student learning was not only deeper on a cognitive level, but also allowed students to understand their own roles and responsibilities in responding to natural hazard threats. The combination of both is explored through the use of an online questionnaire (n = 176) in which the impact of the learning on students and their families are explored. The classroom and individual learning activities’ impact on student efficacy are discussed alongside the results from the questionnaire. Findings included support for prior assumptions about the impact of school-based learning on the family with regard to disaster preparedness as well as deeper cognition regarding the risks and increased self-efficacy in students. The implications for these findings and their role in transforming learning to enhance community resilience that starts with the family are discussed with the door to future research nudged open. | Grade B |
| Taguchi K, Matsoso P, Driece R, da Silva Nunes T, Soliman A, Tangcharoensathien V. Effective infodemic management: A substantive article of the pandemic accord. JMIR infodemiology. 2023 Sep 20;3:e51760. | Social media has proven to be valuable for disseminating public health information during pandemics. However, the circulation of misinformation through social media during public health emergencies, such as the SARS (severe acute respiratory syndrome), Ebola, and COVID-19 pandemics, has seriously hampered effective responses, leading to negative consequences. Intentionally misleading and deceptive fake news aims to harm organizations and individuals. To effectively respond to misinformation, governments should strengthen the management of an "infodemic," which involves monitoring the impact of infodemics through social listening, detecting signals of infodemic spread, mitigating the harmful effects of infodemics, and strengthening the resilience of individuals and communities. The global spread of misinformation requires multisectoral collaboration, such as researchers identifying leading sources of misinformation and superspreaders, media agencies identifying and debunking misinformation, technology platforms reducing the distribution of false or misleading posts and guiding users to health information from credible sources, and governments disseminating clear public health information in partnership with trusted messengers. Additionally, fact-checking has room for improvement through the use of automated checks. Collaboration between governments and fact-checking agencies should also be strengthened via effective and timely debunking mechanisms. Though the Intergovernmental Negotiating Body (INB) has yet to define the term "infodemic," Article 18 of the INB Bureau's text, developed for the Pandemic Accord, encompasses a range of actions aimed at enhancing infodemic management. The INB Bureau continues to facilitate evidence-informed discussion for an implementable article on infodemic management. | Grade B |

**Considering inclusion and equity**

| **Reference** | **Abstract** | **GRADE** |
| --- | --- | --- |
| Galea S, Ahern J, Resnick H, Kilpatrick D, Bucuvalas M, Gold J, Vlahov D. Psychological sequelae of the September 11 terrorist attacks in New York City. New England journal of medicine. 2002 Mar 28;346(13):982-7. | Background: The scope of the terrorist attacks of September 11, 2001, was unprecedented in the United States. We assessed the prevalence and correlates of acute post-traumatic stress disorder (PTSD) and depression among residents of Manhattan five to eight weeks after the attacks. Methods: We used random-digit dialing to contact a representative sample of adults living south of 110th Street in Manhattan. Participants were asked about demographic characteristics, exposure to the events of September 11, and psychological symptoms after the attacks. Results: Among 1008 adults interviewed, 7.5 percent reported symptoms consistent with a diagnosis of current PTSD related to the attacks, and 9.7 percent reported symptoms consistent with current depression (with "current" defined as occurring within the previous 30 days). Among respondents who lived south of Canal Street (i.e., near the World Trade Center), the prevalence of PTSD was 20.0 percent. Predictors of PTSD in a multivariate model were Hispanic ethnicity, two or more prior stressors, a panic attack during or shortly after the events, residence south of Canal Street, and loss of possessions due to the events. Predictors of depression were Hispanic ethnicity, two or more prior stressors, a panic attack, a low level of social support, the death of a friend or relative during the attacks, and loss of a job due to the attacks. Conclusions: There was a substantial burden of acute PTSD and depression in Manhattan after the September 11 attacks. Experiences involving exposure to the attacks were predictors of current PTSD, and losses as a result of the events were predictors of current depression. In the aftermath of terrorist attacks, there may be substantial psychological morbidity in the population. | Grade B |
| Norris, F. H., Stevens, S. P., Pfefferbaum, B., Wyche, K. F., & Pfefferbaum, R. L. (2008). Community resilience as a metaphor, theory, set of capacities, and strategy for disaster readiness. American Journal of Community Psychology, 41(1-2), 127-150. | Communities have the potential to function effectively and adapt successfully in the aftermath of disasters. Drawing upon literatures in several disciplines, we present a theory of resilience that encompasses contemporary understandings of stress, adaptation, wellness, and resource dynamics. Community resilience is a process linking a network of adaptive capacities (resources with dynamic attributes) to adaptation after a disturbance or adversity. Community adaptation is manifest in population wellness, defined as high and non-disparate levels of mental and behavioral health, functioning, and quality of life. Community resilience emerges from four primary sets of adaptive capacities--Economic Development, Social Capital, Information and Communication, and Community Competence--that together provide a strategy for disaster readiness. To build collective resilience, communities must reduce risk and resource inequities, engage local people in mitigation, create organizational linkages, boost and protect social supports, and plan for not having a plan, which requires flexibility, decision-making skills, and trusted sources of information that function in the face of unknowns. | Grade B |
| Patel V, Araya R, Chatterjee S, Chisholm D, Cohen A, De Silva M, Hosman C, McGuire H, Rojas G, Van Ommeren M. Treatment and prevention of mental disorders in low-income and middle-income countries. The Lancet. 2007 Sep 15;370(9591):991-1005. | We review the evidence on effectiveness of interventions for the treatment and prevention of selected mental disorders in low-income and middle-income countries. Depression can be treated effectively in such countries with low-cost antidepressants or with psychological interventions (such as cognitive-behaviour therapy and interpersonal therapies). Stepped-care and collaborative models provide a framework for integration of drug and psychological treatments and help to improve rates of adherence to treatment. First-generation antipsychotic drugs are effective and cost effective for the treatment of schizophrenia; their benefits can be enhanced by psychosocial treatments, such as community-based models of care. Brief interventions delivered by primary-care professionals are effective for management of hazardous alcohol use, and pharmacological and psychosocial interventions have some benefits for people with alcohol dependence. Policies designed to reduce consumption, such as increased taxes and other control strategies, can reduce the population burden of alcohol abuse. Evidence about the efficacy of interventions for developmental disabilities is inadequate, but community-based rehabilitation models provide a low-cost, integrative framework for care of children and adults with chronic mental disabilities. Evidence for mental health interventions for people who are exposed to conflict and other disasters is still weak—especially for interventions in the midst of emergencies. Some trials of interventions for prevention of depression and developmental delays in low-income and middle-income countries show beneficial effects. Interventions for depression, delivered in primary care, are as cost effective as antiretroviral drugs for HIV/AIDS. The process and effectiveness of scaling up mental health interventions has not been adequately assessed. Such research is needed to inform the continuing process of service reform and innovation. However, we recommend that policymakers should act on the available evidence to scale up effective and cost-effective treatments and preventive interventions for mental disorders. | Grade B |
| Sampugnaro R, Santoro P. The pandemic crisis, Italian municipalities, and community resilience. Partecipazione e conflitto. 2021 Jun 24;14(1):283-301. | The pandemic caused by Covid-19 has tested the resilience of public institutions, already burdened by a deep and complex crisis (political, economic, managerial). This crisis has revealed a discrepancy between the needs expressed by the community and the solutions adopted to satisfy them. This has been accompanied by a progressive worsening of decision-making efficiency and weak implementation capacity in a context of increasing environmental uncertainty. It is in local institutions, in particular, that the greatest problems are revealed, because of many endemic negative factors: political fragmentation, reduced economic resources, new forms of poverty. Against the background of this scenario, our study aims to analyze the reaction of local institutions to the pandemic crisis by looking at both welfare and communication services. The objective is to identify key features in understanding the resilience of municipalities. In other words, their ability to react and adapt to change, which is essential not only to deal with emergencies, such as the pandemic, but also to make the institution itself sustainable. Our interest is focused on a specific dimension of the resilience of the municipalities, related to collaboration with the third sector. The pandemic has shown that the continuous activism of non-profit organizations has allowed for the continuation of many so-called "ordinary" services, as well as the launch of several initiatives aimed at alleviating other social problems. The research has, first of all, an exploratory character that befits a new and still ongoing phenomenon. The basic questions concern the production of local welfare policies by municipalities. The data show different levels of "interventism" and different modes of communication. On this latter point, we observe the presence of significant attention-seeking among Mayors as community builders able, on the one hand, to reinforce the spirit of solidarity and, on the other, to uphold respect for the rules. On the services side, three main models of response to the pandemic emerge, two of which refer to the public-private relationship in local welfare policies. Findings suggest that these different reactions will have consequences in the immediate future for the management of the pandemic crisis (still ongoing). Specifically, the tendency is to employ a management of services based on partnership-model, which means that public-private collaboration is a pillar of local welfare. This seems to entail a greater legitimacy for individuals or associations to participate in the formulation and implementation of policies. | Grade B |
| Petrun Sayers EL, Anthony KE, Tom A, Kim AY, Armstrong C. ‘We will rise no matter what’: community perspectives of disaster resilience following Hurricanes Irma and Maria in Puerto Rico. Journal of Applied Communication Research. 2023 Mar 4;51(2):126-45. | Category 4 Hurricane Maria made landfall in Puerto Rico on 20 September 2017 and ploughed across the territory with sustained winds of 155 mph. Just two weeks earlier, category 5 Hurricane Irma had struck the island already damaging critical infrastructure making Hurricane Maria even more devasting. The hurricanes caused catastrophic damage, resulting in the largest and longest response to a domestic disaster in the history of the United States. This paper explores the recovery process in Puerto Rico using a community resilience lens. The study examines narratives, the media environment, trusted sources, and information preferences following the crisis. Community workshops, interviews, and focus groups reveal indicators of resilience in Puerto Rico alongside areas for improvement. Theoretical contributions discuss the role of identity, sense of place, and the impact of culture on community resilience. Practical contributions touch on messaging, acknowledging infrastructure vulnerabilities, and the importance of strengthening community relationships. | Grade C |
| Takahashi K, Kodama M, Gregorio Jr ER, Tomokawa S, Asakura T, Waikagul J, Kobayashi J. School Health: an essential strategy in promoting community resilience and preparedness for natural disasters. Global health action. 2015 Dec 1;8(1):29106. | Background: The Third UN World Conference on Disaster Risk Reduction recommended the implementation of the Sendai Framework for Disaster Risk Reduction 2015-2030, which aims to achieve substantial risk reduction and to avoid various disaster-associated losses, including human lives and livelihoods, based on the lessons from the implementation of the Hyogo framework. However, the recommendations did not lay enough stress on the school and the Safe School Concept, which are the core components of a disaster response. Objective: To raise the issue of the importance of schools in disaster response. Results: For human capacity building to avoid the damage caused by natural disasters, we should focus on the function of schools in the community and on school health framework. Schools perform a range of functions, which include being a landmark place for evacuation, acting as a participatory education hub among communities (students are usually from the surrounding communities), and being a sustainable source of current disaster-related information. In 2007, the Bangkok Action Agenda (BAA) on school education and disaster risk reduction (DRR) recommended the integration of DRR into education policy development, the enhancement of participatory mechanisms to improve DRR education, and the extension of DRR education from schools to communities. Based on our discussion and the recommendations of the BAA, we suggest that our existing challenges are to construct a repository of disaster-related lessons, develop training materials based on current information drawn from previous disasters, and disseminate the training to schools and communities. Conclusions: Schools linked with school health can provide good opportunities for DRR with a focus on development of school health policy and a community-oriented participatory approach. | Grade C |
| Veil SR, Bishop BW. Opportunities and challenges for public libraries to enhance community resilience. Risk analysis. 2014 Apr;34(4):721-34. | This study bridges a gap between public library and emergency management policy versus practice by examining the role of public libraries in the community resource network for disaster recovery. Specifically, this study identifies the opportunities and challenges for public libraries to fulfill their role as a FEMA-designated essential community organization and enhance community resilience. The results indicate there are several opportunities for libraries to enhance community resilience by offering technology resources and assistance; providing office, meeting, and community living room space; serving as the last redundant communication channel and a repository for community information and disaster narratives; and adapting or expanding services already offered to meet the changing needs of the community. However, libraries also face challenges in enhancing community resilience, including the temptation to overcommit library capacity and staff capability beyond the library mission and a lack of long-term disaster plans and collaboration with emergency managers and government officials. Implications for library and emergency management practice and crisis research are discussed. | Grade C |
| Lee PC. From community engagement to community resilience: the evolving role of public libraries. Public Library Quarterly. 2024 May 3;43(3):339-66. | This study examines the role of public libraries in promoting community resilience and sustainability during times of crisis. It fills a research gap by investigating the relationship between library services, community engagement, and long-term resilience within the context of disaster recovery. Through a comprehensive analysis of literature and a case study of Kaohsiung Public Library in Taiwan, the study establishes a robust association between libraries and community resilience. The study emphasizes the importance of public libraries as agents of community resilience and advocates for a comprehensive approach to address community needs during crises. | Grade C |

**Community engagement and feedback**

| **Reference** | **Abstract** | **GRADE** |
| --- | --- | --- |
| Anthony KE, Venette SJ, Pyle AS, Boatwright BC, Reif-Stice CE. The role of social media in enhancing risk communication and promoting community resilience in the midst of a disaster. InRisk communication and community resilience 2019 May 28 (pp. 165-178). Routledge. | The importance of engendering community resilience after a disaster event cannot be overstated. In this chapter, we argue that the strategic design of risk communication messages is critical for communities affected by crises. In the past, many communication practitioners employed the single shot, top-down approach for constructing messages, especially during crises. Given growing recognition of the ineffectiveness of this model, scholars have become more attentive to the role of message design in conveying risk to stakeholders. Specifically, through the lens of the message convergence framework, we argue the need for a multiple messages approach that targets diverse groups of people through various channels, including social media. Not only should multiple messages be present, but response organizations must work with each other to ensure consistency of message content, as the convergence or overlap of information promotes individual decision-making during times of risk and uncertainty. Additionally, the integration of social media platforms into crisis response plans is imperative. Social media not only are easily accessible and already widely used by much of the public, but in addition to risk message dissemination, social media platforms can allow for the mapping of disasters, which enables responders to better understand the location and distribution of areas most affected by an event. We conclude with recommendations for employing social media in disaster communication research. | Grade C |
| Cabinet Office (2022) The UK Government Resilience Framework. Cabinet Office, London. https://www.gov.uk/government/publications/the-uk-government-resilience-framework (Accessed 1 November 2024) | The professionalism and commitment of the people who contribute to the UK’s resilience is extraordinary and we have a well established framework for civil protection in the UK. But the last few years have exposed the need to build on these strong foundations and strengthen our resilience in order to better prevent, mitigate, respond to and recover from the risks facing the nation. That is why the UK Government committed, in the Integrated Review[footnote 1], to a new Resilience Strategy. The framework is the first articulation of how the UK Government will deliver on a new strategic approach to resilience. It is based on three core principles: A developed and shared understanding of the civil contingencies risks we face is fundamental; Prevention rather than cure wherever possible: a greater emphasis on preparation and prevention; and Resilience is a ‘whole of society’ endeavour, so we must be more transparent and empower everyone to make a contribution. This framework focuses on the foundational building blocks of resilience, setting out the plan to 2030 to strengthen the frameworks, systems and capabilities which underpin the UK’s resilience to all civil contingencies risks. | Grade C |
| Hu G, Qiu W. From guidance to practice: Promoting risk communication and community engagement for prevention and control of coronavirus disease (COVID‐19) outbreak in China. Journal of Evidence‐Based Medicine. 2020 May;13(2):168-72. | Integrating risk communication and community engagement into the national public health emergency response is crucial. Considering the difficulties and challenges faced by China in the prevention and control of coronavirus disease (COVID-19) and based on interim guidelines from the World Health Organization, this article makes several recommendations addressing the outbreak in China. These include improvements in the internal governmental risk communication systems, enhancing the coordination between internal and partner governmental emergency management, and promoting public communication in response to societal concerns. Regarding these recommendations, we emphasize community engagement in joint prevention and control, confronting uncertainty and countering rumors effectively, and strengthening international cooperation and evidence-based decision making for prevention and control measures. | Grade C |
| Sampugnaro R, Santoro P. The pandemic crisis, Italian municipalities, and community resilience. Partecipazione e conflitto. 2021 Jun 24;14(1):283-301. | The pandemic caused by Covid-19 has tested the resilience of public institutions, already burdened by a deep and complex crisis (political, economic, managerial). This crisis has revealed a discrepancy between the needs expressed by the community and the solutions adopted to satisfy them. This has been accompanied by a progressive worsening of decision-making efficiency and weak implementation capacity in a context of increasing environmental uncertainty. It is in local institutions, in particular, that the greatest problems are revealed, because of many endemic negative factors: political fragmentation, reduced economic resources, new forms of poverty. Against the background of this scenario, our study aims to analyze the reaction of local institutions to the pandemic crisis by looking at both welfare and communication services. The objective is to identify key features in understanding the resilience of municipalities. In other words, their ability to react and adapt to change, which is essential not only to deal with emergencies, such as the pandemic, but also to make the institution itself sustainable. Our interest is focused on a specific dimension of the resilience of the municipalities, related to collaboration with the third sector. The pandemic has shown that the continuous activism of non-profit organizations has allowed for the continuation of many so-called "ordinary" services, as well as the launch of several initiatives aimed at alleviating other social problems. The research has, first of all, an exploratory character that befits a new and still ongoing phenomenon. The basic questions concern the production of local welfare policies by municipalities. The data show different levels of "interventism" and different modes of communication. On this latter point, we observe the presence of significant attention-seeking among Mayors as community builders able, on the one hand, to reinforce the spirit of solidarity and, on the other, to uphold respect for the rules. On the services side, three main models of response to the pandemic emerge, two of which refer to the public-private relationship in local welfare policies. Findings suggest that these different reactions will have consequences in the immediate future for the management of the pandemic crisis (still ongoing). Specifically, the tendency is to employ a management of services based on partnership-model, which means that public-private collaboration is a pillar of local welfare. This seems to entail a greater legitimacy for individuals or associations to participate in the formulation and implementation of policies. | Grade C |
| Schiavo R. Health communication: From theory to practice. John Wiley & Sons; 2013 Oct 7. | COVID-19 has changed our world in many ways. Among others, the pandemic and its disproportionate burden among populations that have been socially and economically marginalized, or experience other kinds of vulnerability or disadvantage, are presenting organizations and their leaders with an opportunity to reflect on key drivers of global health efforts, and to address health and social issues that contribute to local inequities. Increasingly, ‘community engagement’ has become a buzzword among a variety of health and human rights organizations and government agencies. It’s also the focus of new resources, and capacity building and training efforts. With the increasing use of this term, there are also some confusions on what ‘community engagement’ actually is. This renewed emphasis on the centrality of communities is important for the future of healthcare and global health, and calls for a shared understanding of the actual meaning of ‘community engagement.’ | Grade C |
| Takahashi K, Kodama M, Gregorio Jr ER, Tomokawa S, Asakura T, Waikagul J, Kobayashi J. School Health: an essential strategy in promoting community resilience and preparedness for natural disasters. Global health action. 2015 Dec 1;8(1):29106. | Background: The Third UN World Conference on Disaster Risk Reduction recommended the implementation of the Sendai Framework for Disaster Risk Reduction 2015-2030, which aims to achieve substantial risk reduction and to avoid various disaster-associated losses, including human lives and livelihoods, based on the lessons from the implementation of the Hyogo framework. However, the recommendations did not lay enough stress on the school and the Safe School Concept, which are the core components of a disaster response. Objective: To raise the issue of the importance of schools in disaster response. Results: For human capacity building to avoid the damage caused by natural disasters, we should focus on the function of schools in the community and on school health framework. Schools perform a range of functions, which include being a landmark place for evacuation, acting as a participatory education hub among communities (students are usually from the surrounding communities), and being a sustainable source of current disaster-related information. In 2007, the Bangkok Action Agenda (BAA) on school education and disaster risk reduction (DRR) recommended the integration of DRR into education policy development, the enhancement of participatory mechanisms to improve DRR education, and the extension of DRR education from schools to communities. Based on our discussion and the recommendations of the BAA, we suggest that our existing challenges are to construct a repository of disaster-related lessons, develop training materials based on current information drawn from previous disasters, and disseminate the training to schools and communities. Conclusions: Schools linked with school health can provide good opportunities for DRR with a focus on development of school health policy and a community-oriented participatory approach. | Grade C |
| Yeo J, Knox CC, Jung K. Unveiling cultures in emergency response communication networks on social media: Following the 2016 Louisiana floods. Quality & quantity. 2018 Mar;52:519-35. | While culture in emergency management has gained attention from the field of risk communication, few have systemically dealt with the nuances of general culture involved in the formation and differentiation of risk communication. To fill this gap, this research aims to first examine cultural nuances from the 2016 Louisiana flood response by primarily focusing on communications embedded in social media. The results from social network analysis and content analysis highlight that the flood response communication had strong cultural characteristics, highlighting the notion that of the cultures in Louisiana-faith-based, local authority, and nonprofits-were the prominent cultural responders in the flood response communication. In particular, cultural similarity in both intra/inter group response communication was observed, with each communication group comprising actors who shared a common cultural background and spoke similar keywords. | Grade C |
| Zhang L, Zhao J, Liu J, Chen K. Community disaster resilience in the COVID-19 outbreak: insights from Shanghai’s experience in China. Risk management and healthcare policy. 2021 Jan 5:3259-70. | Purpose: Communities are central to the practice of public health emergency preparedness and response. This article mainly focuses on COVID-19 and discusses the formation and structure of community disaster resilience, which is an effective method for coping with such a public health emergency. Methods: Based on the management of the COVID-19 outbreak in China, this article uses Shanghai's experience to illustrate how a community disaster resilience was formed for risk management. Resorting to the analytical framework of risk city, principles of community disaster resilience are given. Results: Four actions can be recommended based on Shanghai's experience: 1) Applying a vulnerability analysis matrix for targeted risk governance, 2) empowering volunteer groups for emergency response, 3) policy and action for public health emergency prevention, and 4) risk communication for uncertainty-oriented planning. Conclusion: Shanghai's experience offers a reference to tackle the COVID-19 at the global level. The COVID-19 outbreak highlights that humans still face various unpredictable health risks in the future. Forming a connection-based resilience at the community level is an effective way to risk management. | Grade B |

**Theme 2: Strategies and interventions to enhance community resilience in health emergency communication**

**Facilitating community structures as channels for communication**

| **Reference** | **Abstract** | **GRADE** |
| --- | --- | --- |
| An J, Kwak H, Qureshi HM, Weber I. Precision public health campaign: delivering persuasive messages to relevant segments through targeted advertisements on social media. JMIR formative research. 2021 Sep 24;5(9):e22313. | Although established marketing techniques have been applied to design more effective health campaigns, more often than not, the same message is broadcasted to large populations, irrespective of unique characteristics. As individual digital device use has increased, so have individual digital footprints, creating potential opportunities for targeted digital health interventions. We propose a novel precision public health campaign framework to structure and standardize the process of designing and delivering tailored health messages to target particular population segments using social media–targeted advertising tools. Our framework consists of five stages: defining a campaign goal, priority audience, and evaluation metrics; splitting the target audience into smaller segments; tailoring the message for each segment and conducting a pilot test; running the health campaign formally; and evaluating the performance of the campaigns. We have demonstrated how the framework works through 2 case studies. The precision public health campaign framework has the potential to support higher population uptake and engagement rates by encouraging a more standardized, concise, efficient, and targeted approach to public health campaign development. | Grade B |
| Bear L, James D, Simpson N, Alexander E, Bazambanza C, Bhogal JK, Bowers R, Cannell F, Lohiya A, Koch I, Lenhard J. A right to care: the social foundations of recovery from Covid-19. http://eprints.lse.ac.uk/id/eprint/107060 | This report presents key findings from a 6-month ethnographic study on the impact of the Covid-19 pandemic on disadvantaged households and communities across the UK conducted by anthropologists from the London School of Economics, and associates. This research involved in-depth interviews and multiple surveys with people across communities in the UK, with particular focus on a number of case studies of intersecting disadvantage. Crucially, our research has found that Government policy can improve adherence to restrictions and reduce the negative impacts of the pandemic on disadvantaged communities by placing central importance on communities, social networks and households to the economy and social life. This would be the most effective way to increase public trust and adherence to Covid-19 measures, because it would recognise the suffering that communities have experienced and would build policy on the basis of what is most important to people - the thriving of their families and communities. | Grade B |
| Boyce MR, Katz R. Community health workers and pandemic preparedness: current and prospective roles. Frontiers in public health. 2019 Mar 26;7:62. | Despite the importance of community health workers (CHWs) to health systems in resource-constrained environments, relatively little has been written about their contributions to pandemic preparedness. In this perspective piece, we draw from the response to the 2014 Ebola and 2015 Zika epidemics to review examples whereby CHWs contributed to health security and pandemic preparedness. CHWs promoted pandemic preparedness prior to the epidemics by increasing the access to health services and products within communities, communicating health concepts in a culturally appropriate fashion, and reducing the burdens felt by formal healthcare systems. During the epidemics, CHWs promoted pandemic preparedness by acting as community-level educators and mobilizers, contributing to surveillance systems, and filling health service gaps. Acknowledging the success CHWs have had in these roles and in previous interventions, we propose that the cadre may be better engaged in pandemic preparedness in the future. Some practical strategies for achieving this include training and using CHWs to communicate One Health information to at-risk communities prior to outbreaks, pooling them into a reserve health corps to be used during public health emergencies, and formalizing agreements and strategies to promote the early engagement of CHWs in response actions. Recognizing that CHWs already play a role in pandemic preparedness, we feel that expanding the roles and responsibilities of CHWs represents a practical means of improving pandemic and community-level resilience. | Grade B |
| Centre for Disease Control and Prevention, 2021. Access and Functional Needs Toolkit Integrating a Community Partner Network to Inform Risk Communication Strategies https://www.cdc.gov/readiness/media/pdfs/CDC_Access_and_Functional_Needs_Toolkit_March2021.pdf | Communicating public health information is an essential element of emergency preparedness and response. Timely and effective messages can help minimize people’s risk or vulnerability. For example, public health messages can inform people how to stay safe, take shelter, or evacuate. Emergency management officials, public health professionals, and other stakeholders achieve effective risk communication by using preparedness planning and by developing messages for the whole community. This includes individuals who may be at greater risk or who need additional assistance because of access and functional needs. What are access and functional needs? For the purposes of this toolkit, the term “access and functional needs” refers to individuals with and without disabilities, who may need additional assistance because of any condition (temporary or permanent) that may limit their ability to act in an emergency. Individuals with “access and functional needs” do not require any kind of diagnosis or specific evaluation.2 These may include but are not limited to individuals with disabilities, individuals with limited English proficiency, individuals with limited access to transportation, individuals with limited access to financial resources, older adults, and others deemed “at risk” by the Pandemic and All-Hazards Preparedness and Advancing Innovation Act (PAHPAIA) or the Secretary of Health and Human Services. | Grade D |
| Fernandes-Jesus M, Mao G, Ntontis E, Cocking C, McTague M, Schwarz A, Semlyen J, Drury J. More than a COVID-19 response: sustaining mutual aid groups during and beyond the pandemic. Frontiers in psychology. 2021 Oct 20;12:716202. | Mutual aid groups have been an indispensable part of the public response to the COVID-19 pandemic. They have provided many forms of support, in particular grocery shopping which has enabled people to self-isolate if required. While community solidarity during emergencies and disasters is common, previous studies have shown that such solidarity behaviors tend to decline over time, even when needs remain high. In this study, we address how mutual aid groups can be sustained over time in the context of the COVID-19 pandemic. We conducted 32 interviews with organizers of COVID-19 mutual aid and community support groups in the United Kingdom between September 2020 and January 2021. Based on a reflexive thematic analysis, we identified several community and group level experiences and strategies that were related to sustained participation in COVID-19 mutual aid groups. Meeting community needs over time with localized action and resources and building trust and community-based alliances were foundational elements in the COVID-19 mutual aid groups. Group processes strategies, such as a culture of care and support and regular group meetings, were used to help to sustain involvement. Some experiences resulting from participation in COVID-19 mutual aid groups were also related to sustained participation, including positive emotions (e.g., joy, pride), well-being and sense of efficacy, and an increasing sense of local community belonging and cohesion. Based on these findings, we propose four practical recommendations for sustaining mutual aid groups to assist public engagement with protective behaviors in the COVID-19 pandemic and beyond. We recommend providing practical and financial support to COVID-19 mutual aid groups; to mobilize the knowledge and the experiences acquired by COVID-19 mutual aid groups for developing programs and interventions for addressing the medium and long-term impacts of COVID-19; to prioritize community-level interventions; and to recognize the role of group processes as these have the potential to lead to long-term community responses. These approaches will be key for ensuring that communities effectively recover from the COVID-19 pandemic. | Grade B |
| Kamal A, Bear L. Community Champions policy: key principles and strategic implications for recovery from Covid-19. https://eprints.lse.ac.uk/122478/1/Bear_community_champions_policy_published.pdf | The Department for Levelling Up Housing and Communities (DLUHC, formerly the Ministry of Housing Communities and Local Government) funded the Community Champions programme to provide a framework which aligns key messages at a national and local level during a national emergency. The programme, initiated in March 2021, has amplified and supported the social infrastructures of mutual aid and volunteering that emerged during the first wave of the Covid-19 pandemic. It is a success story of central government pandemic policy because it articulated well with local level efforts, thereby strengthening and sustaining regional capacities to deal with Covid-19. Three local authority areas (names not reported to maintain participant anonymity) were selected for a spotlight evaluation as they were super-diverse areas (or areas with a high level of diversity of social groups across dimensions of race, class, gender, ethnicity and religion and a high diversity of social positioning within groups [1]), high on the indices of multiple deprivation, and were initiating Community Champion programmes for the first time. Data was collected over three time points to explore the experience of initiating, implementing, and maintaining the programme. A comparative analysis of delivery of community-led interventions through shorter term surge funding of Voluntary Community Social Enterprise (VCSE) delivery partners is also reported. | Grade B |
| O'Dwyer E, Beascoechea‐Seguí N, Souza LG. The amplifying effect of perceived group politicization: Effects of group perceptions and identification on anxiety and coping self‐efficacy among members of UK COVID‐19 mutual aid groups. Journal of community & applied social psychology. 2022 May;32(3):423-37. | Mutual aid groups developed and mobilized in communities across the UK and globally at the outset of the pandemic in order to support vulnerable community members with practical assistance and emotional support, with some understanding their work in political terms. This study adopted a "social cure" lens to investigate the effects of group identification and group perceptions on anxiety and coping self-efficacy among members of UK Covid-19 mutual aid groups. Survey data were collected from self-identified members of these groups (N = 844) during the initial period of "lockdown" restrictions in April - May 2020. Correlational analyses showed that identification with the mutual aid group was linked to more positive group perceptions and better self-reported psychological outcomes. Perceived group politicization showed the reverse pattern. Mixed support for the "social cure" model was evident; the effect of group identification on coping self-efficacy (but not anxiety) was serially mediated by perceived support and collective efficacy. Perceived group politicization was a significant moderator, seeming to amplify the indirect effect of group identification on coping self-efficacy via perceived support. Results are discussed in light of previous empirical work on the social cure and Covid-19 mutual aid groups. Please refer to the Supplementary Material section to find this article's Community and Social Impact Statement. | Grade B |
| Wijesinghe MS, Gunawardana BM, Weerasinghe WM, Karunarathne SA, Vithana VC, Rajapaksha RM, Batuwanthudawe R, Karunapema RP. Empowering communities during the COVID-19 pandemic through mothers’ support groups: evidence from a community engagement initiative in Sri Lanka. Global Health: Science and Practice. 2023 Apr 28;11(2). | Community empowerment has previously been used to mitigate the effects of health emergencies, such as outbreaks of Ebola virus disease and Zika virus. In Sri Lanka, mothers' support groups (MSGs) aim to promote community health, well-being, and nutrition practices through community engagement. With the emergence of the COVID-19 pandemic, MSGs were mobilized by the Health Promotion Bureau in response to the pandemic. Key activities MSGs engaged in during the COVID-19 response included establishing communication networks, creating a supportive environment for preventive behaviors, organizing vaccination clinics, distributing essential food and medicine, organizing recreational activities, promoting home gardening, and monitoring community activities. We examine how these community-based empowerment initiatives successfully assisted in the pandemic response. Health officials can help to build more resilient communities that are better prepared for future health emergencies by valuing the community-level methods used in overcoming COVID-19 and further strengthening the skills of community group members. | Grade B |
| Xie L, Pinto J, Zhong B. Building community resilience on social media to help recover from the COVID-19 pandemic. Computers in Human Behavior. 2022 Sep 1;134:107294. | Facing the Covid outbreaks, public health researchers share a consensus that community resilience should be maintained and strengthened because it helps mitigate the physical and emotional tolls on individuals and communities. One way to achieve the goal is to build and strengthen community resilience through social media. However, social media's role in building community resilience has been poorly understood from a behavioral perspective. Guiding by uses and gratification theory and the coping literature, we build a model to examine how social media behaviors may influence community members' perceived community resilience, providing a "bottom-up" voice to deepen our understanding of community resilience and its implications for public health. The results shows that community members' social media engagement was significantly associated with their perceived community resilience. While helping others on social media led people to perceive their communities as less resilient, the use of social media for social support helped foster social capital, leading to more perceived resilience at the collective level. Overall, social media use played important roles in shaping people's perception of community resilience, helping community members and organizations evaluate their strengths and weaknesses, and make improvement to better address future challenges in the times of global disasters. | Grade B |

**Respecting personal and private boundaries in health communication**

| **Reference** | **Abstract** | **GRADE** |
| --- | --- | --- |
| Forsberg JT, Schultz JH. Educational and psychosocial support for conflict-affected youths: The effectiveness of a school-based intervention targeting academic underachievement. International Journal of School & Educational Psychology. 2023 Apr 3;11(2):145-66. | This study investigated the effect of a school-based and teacher-led psychosocial intervention that targeted academic underachievement among conflict-affected youths. We hypothesized that participants in the intervention would experience improved school functioning and reduced levels of stress-related symptoms after the intervention, in comparison to a control group. The study was carried out in Gaza, employed a randomized control trial design, and included 300 students between 9 and16 years of age. Students that completed the intervention reported improvement in all the domains measured after participation: better school functioning, a reduced level of stress-related symptoms, as well as long-term effects in the ability to self-regulate negative emotions, self-efficacy, better study skills, and a higher academic performance. The long-term effects were observed five months after the intervention was carried out. The implications for the implementation of psychosocial support for conflict-affected youth are discussed. | Grade B |
| Hanson K, O’Dwyer E, Chaudhuri S, Silva Souza LG, Vandrevala T. Mitigating the identity and health threat of COVID-19: Perspectives of middle-class South Asians living in the UK. Journal of Health Psychology. 2022 Aug;27(9):2147-60. | The recognition and representation of BAME community as ‘high risk’ of Covid-19 in the UK presents both a health and an identity threat to this ethnic group. This study employed thematic analysis to explore response to these threats as related by a sample of 13 middle class members of the South Asian community. This work advances both health and identity psychological theory by recognising the affinity between expressions of health efficacy and identity. Our findings identify South Asian intragroup stigmatisation and commonalities that have implications for the promotion of health behaviour and health communications for minority groups. | Grade B |
| Heris CL, Kennedy M, Graham S, Bennetts SK, Atkinson C, Mohamed J, Woods C, Chennall R, Chamberlain C. Key features of a trauma-informed public health emergency approach: A rapid review. Frontiers in public health. 2022 Nov 28;10:1006513. | COVID-19 is a major threat to public safety, and emergency public health measures to protect lives (e.g., lockdown, social distancing) have caused widespread disruption. While these measures are necessary to prevent catastrophic trauma and grief, many people are experiencing heightened stress and fear. Public health measures, risks of COVID-19 and stress responses compound existing inequities in our community. First Nations communities are particularly at risk due to historical trauma, ongoing socio-economic deprivation, and lack of trust in government authorities as a result of colonization. The objective of this study was to review evidence for trauma-informed public health emergency responses to inform development of a culturally-responsive trauma-informed public health emergency framework for First Nations communities. We searched relevant databases from 1/1/2000 to 13/11/2020 inclusive, which identified 40 primary studies (and eight associated references) for inclusion in this review. Extracted data were subjected to framework and thematic synthesis. No studies reported evaluations of a trauma-informed public health emergency response. However, included studies highlighted key elements of a "trauma-informed lens," which may help to consider implications, reduce risks and foster a sense of security, wellbeing, self- and collective-efficacy, hope and resilience for First Nations communities during COVID-19. We identified key elements for minimizing the impact of compounding trauma on First Nations communities, including: a commitment to equity and human rights, cultural responsiveness, good communication, and positive leadership. The six principles guiding trauma-informed culturally-responsive public health emergency frameworks included: (i) safety, (ii) empowerment, (iii) holistic support, (iv) connectedness and collaboration, (v) compassion and caring, and (vi) trust and transparency in multi-level responses, well-functioning social systems, and provision of basic services. These findings will be discussed with First Nations public health experts, together with data on the experiences of First Nations families and communities during COVID-19, to develop a trauma-integrated public health emergency response framework or "lens" to minimize compounding trauma for First Nations communities. | Grade B |
| Miller-Karas, E. Building Resilience to Trauma: The Trauma and Community Resiliency Models, 2nd edition. ed. Routledge, Abingdon, Oxon; 2023 New York, NY. | During and after a traumatic experience, survivors experience a cascade of physical, emotional, cognitive, behavioral, relational, and spiritual responses that can make them feel unbalanced and threatened. The second edition of Building Resilience to Trauma explains common responses from a biological perspective, reframing the human experience from one of shame and pathology to one of hope and biology. Using two evidence-informed models of intervention that are trauma-informed and resiliency-informed—the Community Resiliency Model (CRM) and the Trauma Resiliency Model (TRM)—chapters distill complex neuroscience into understandable concepts and lay out a path for fostering short- and long-term healing. CRM develops natural leaders who share wellness skills throughout communities as primary prevention, and TRM focuses on training mental health professionals to reprocess traumatic experiences. Studies have demonstrated that the models’ use leads to significant reductions in depression and anxiety, and both models also lead to increases in well-being. The models restore balance after traumatic experiences and can be used as tools to cultivate well-being across cultures and abilities throughout the lifespan. Program cosponsors have included the United Nations, Emory University's Center for Contemplative Science and Compassion-Based Ethics, the Victims and Survivors Network of Northern Ireland, PACES Connection, the International Transformational Resilience Coalition, the Adventist Disaster Relief Agency International, Wake County School System, and the State of Washington Police Commission. | Grade B |
| Takahashi K, Kodama M, Gregorio Jr ER, Tomokawa S, Asakura T, Waikagul J, Kobayashi J. School Health: an essential strategy in promoting community resilience and preparedness for natural disasters. Global health action. 2015 Dec 1;8(1):29106. | Background: The Third UN World Conference on Disaster Risk Reduction recommended the implementation of the Sendai Framework for Disaster Risk Reduction 2015-2030, which aims to achieve substantial risk reduction and to avoid various disaster-associated losses, including human lives and livelihoods, based on the lessons from the implementation of the Hyogo framework. However, the recommendations did not lay enough stress on the school and the Safe School Concept, which are the core components of a disaster response. Objective: To raise the issue of the importance of schools in disaster response. Results: For human capacity building to avoid the damage caused by natural disasters, we should focus on the function of schools in the community and on school health framework. Schools perform a range of functions, which include being a landmark place for evacuation, acting as a participatory education hub among communities (students are usually from the surrounding communities), and being a sustainable source of current disaster-related information. In 2007, the Bangkok Action Agenda (BAA) on school education and disaster risk reduction (DRR) recommended the integration of DRR into education policy development, the enhancement of participatory mechanisms to improve DRR education, and the extension of DRR education from schools to communities. Based on our discussion and the recommendations of the BAA, we suggest that our existing challenges are to construct a repository of disaster-related lessons, develop training materials based on current information drawn from previous disasters, and disseminate the training to schools and communities. Conclusions: Schools linked with school health can provide good opportunities for DRR with a focus on development of school health policy and a community-oriented participatory approach. | Grade B |
| Vandrevala T, Alidu L, Hendy J, Shafi S, Ala A. ‘It’s possibly made us feel a little more alienated’: How people from ethnic minority communities conceptualise COVID-19 and its influence on engagement with testing. Journal of Health Services Research & Policy. 2022 Apr;27(2):141-50. | Objectives: The cultural beliefs, practices and experiences of ethnic minority groups, alongside structural inequalities and the political economy play a critical but overlooked role in health promotion. This study aimed to understand how ethnic minority groups in the United Kingdom conceptualised COVID-19 and how this influenced engagement in testing. Method: Black (African and Caribbean) and South Asian (Indian, Pakistani and Bangladeshi) community members were purposefully recruited from across the UK. Fifty-seven semi-structured interviews were conducted and analysed using principles of grounded theory. Results: We found that people of Black and South Asian ethnicity conceptualised COVID-19 as a disease that makes them visible to others outside their community and was seen as having more severe risk and suffering worse consequences, resulting in fear, stigmatisation and alienation. Views about COVID-19 were embedded in cultural beliefs, relating to culturally specific ideas around disease, such as ill-health being God’s will. Challenges brought about by the pandemic were conceptualised as one of many struggles, with the saliency of the virus contextualised against life experiences. These themes and others influenced engagement with COVID-19 testing. Testing was less about accessing timely and effective treatment for themselves and more about acting to protect the family and community. Testing symbolised a loss of income, anxiety and isolation, accentuated by issues of mistrust of the system and not being valued, or being treated unfairly. Conclusion: Health communications should focus on counterbalancing the mistrust, alienation and stigmatisation that act as barriers to testing, with trust built using local credible sources. | Grade C |

**Targeting outreach for effective crisis communication**

| **Reference** | **Abstract** | **GRADE** |
| --- | --- | --- |
| Adams RM, Karlin B, Eisenman DP, Blakley J, Glik D. Who participates in the Great ShakeOut? Why audience segmentation is the future of disaster preparedness campaigns. International journal of environmental research and public health. 2017 Nov;14(11):1407. | Background: In 2008, the Southern California Earthquake Center in collaboration with the U.S. Geological Survey Earthquake Hazards Program launched the first annual Great ShakeOut, the largest earthquake preparedness drill in the history of the United States. Materials and Methods: We collected online survey data from 2052 campaign registrants to assess how people participated, whether audience segments shared behavioral patterns, and whether these segments were associated with five social cognitive factors targeted by the ShakeOut campaign. Results: Participants clustered into four behavioral patterns. The Minimal cluster had low participation in all activities (range: 0–39% participation). The Basic Drill cluster only participated in the drop, cover and hold drill (100% participation). The Community-Oriented cluster, involved in the drill (100%) and other interpersonal activities including attending disaster planning meetings (74%), was positively associated with interpersonal communication (β = 0.169), self-efficacy (β = 0.118), outcome efficacy (β = 0.110), and knowledge about disaster preparedness (β = 0.151). The Interactive and Games cluster, which participated in the drill (79%) and two online earthquake preparedness games (53% and 75%), was positively associated with all five social cognitive factors studied. Conclusions: Our results support audience segmentation approaches to engaging the public, which address the strengths and weaknesses of different segments. Offering games may help “gamers” gain competencies required to prepare for disasters. Targeting the highly active Community-Oriented cluster for leadership roles could help build community resilience by encouraging others to become more involved in disaster planning. We propose that the days of single, national education campaigns without local variation should end. | Grade C |
| Council of Europe (2017) New and innovative forms of youth participation in decision-making processes. (Accessed 1 November 2024) https://rm.coe.int/new-and-innovative-forms-of-youth-participation-in-decision-making-pro/1680759e6a | This study of new and innovative forms of youth participation was commissioned by the Council of Europe’s Youth Department. The study focuses on young people’s participation in decision-making processes at national, regional and local levels. The findings and recommendations in the study are intended to inform the Council of Europe youth sector’s future work in this field and its strategic objectives to support young people’s (positive) attitude to influence decisions in democratic processes and increase their involvement in the development of inclusive and peaceful societies. | Grade B |
| National Academies, Policy, Global Affairs, Committee on Science, Public Policy, Committee on Increasing National Resilience to Hazards, Disasters. Disaster resilience: A national imperative. National Academies Press; 2012 Dec 29. | No person or place is immune from disasters or disaster-related losses. Infectious disease outbreaks, acts of terrorism, social unrest, or financial disasters in addition to natural hazards can all lead to large-scale consequences for the nation and its communities. Communities and the nation thus face difficult fiscal, social, cultural, and environmental choices about the best ways to ensure basic security and quality of life against hazards, deliberate attacks, and disasters. Beyond the unquantifiable costs of injury and loss of life from disasters, statistics for 2011 alone indicate economic damages from natural disasters in the United States exceeded $55 billion, with 14 events costing more than a billion dollars in damages each. | Grade B |
| Semmens KA, Carr RH, Maxfield K, Sickler J. CREATE resilience through science, art, and community engagement. Community Science. 2023 Sep;2(3):e2023CSJ000028. | Resilience, specifically community resilience, has a range of definitions but several core elements, including social cohesion and collaboration. Importantly, community-driven goals and approaches tend to be more effective. The CREATE Resilience project centered on co-creating a community vision of resilience, specifically as it relates to natural hazards and climate change by focusing on a positive narrative. By engaging youth, artists, municipal officials and community members in a variety of activities, including surveys, story-gathering and photovoice exhibits, forums, artist-created murals, and ripple effect mapping (REM), the project increased knowledge of weather and climate, risks from local hazards, and strategies for mitigation, while leading the community in thinking about what resilience means. This article describes the project, its use of science, art, and community to co-create a vision of resilience for three communities, the components of engagement and their intent, and the evaluation of impact for participants. As determined through surveys and REM, the CREATE project was effective due to the mixture of art, science and community engagement, which provided a range of opportunities for personal connection and learning related to the science and priorities around hazards and mitigation, helping participants with meaning-making about local hazards and assets, and allowing for a sense of familiarity and interconnectedness. Creating a shared vision of resilience is an effort that engages, connects, and motivates a community around common values and goals, and the approaches implemented through the CREATE project may offer ideas other communities can adopt in efforts to improve resilience. | Grade C |

**Building resilience through communication initiatives**

| **Reference** | **Abstract** | **GRADE** |
| --- | --- | --- |
| Lekas HM, Pahl K, Fuller Lewis C. Rethinking cultural competence: Shifting to cultural humility. Health services insights. 2020 Dec;13:1178632920970580. | Healthcare and social services providers are deemed culturally competent when they offer culturally appropriate care to the populations they serve. While a review of the literature highlights the limited effectiveness of cultural competence training, its value remains largely unchallenged, and it is institutionally mandated as a means of decreasing health disparities and improving quality of care. A plethora of trainings are designed to expose providers to different cultures and expand their understanding of the beliefs, values and behavior thus, achieving competence. Although this intention is commendable, training providers in becoming competent in various cultures presents the risk of stereotyping, stigmatizing, and othering patients and can foster implicit racist attitudes and behaviors. Further, by disregarding intersectionality, cultural competence trainings tend to undermine provider recognition that patients inhabit multiple social statuses that potentially shape their beliefs, values and behavior. To address these risks, we propose training providers in cultural humility, that is, an orientation to care that is based on self-reflexivity, appreciation of patients' lay expertise, openness to sharing power with patients, and to continue learning from one's patients. We also briefly discuss our own cultural humility training. Training providers in cultural humility and abandoning the term cultural competence is a long-awaited paradigm shift that must be advanced. | Grade C |
| Norris, F. H., Stevens, S. P., Pfefferbaum, B., Wyche, K. F., & Pfefferbaum, R. L. (2008). Community resilience as a metaphor, theory, set of capacities, and strategy for disaster readiness. American Journal of Community Psychology, 41(1-2), 127-150. | Communities have the potential to function effectively and adapt successfully in the aftermath of disasters. Drawing upon literatures in several disciplines, we present a theory of resilience that encompasses contemporary understandings of stress, adaptation, wellness, and resource dynamics. Community resilience is a process linking a network of adaptive capacities (resources with dynamic attributes) to adaptation after a disturbance or adversity. Community adaptation is manifest in population wellness, defined as high and non-disparate levels of mental and behavioral health, functioning, and quality of life. Community resilience emerges from four primary sets of adaptive capacities--Economic Development, Social Capital, Information and Communication, and Community Competence--that together provide a strategy for disaster readiness. To build collective resilience, communities must reduce risk and resource inequities, engage local people in mitigation, create organizational linkages, boost and protect social supports, and plan for not having a plan, which requires flexibility, decision-making skills, and trusted sources of information that function in the face of unknowns. | Grade C |
| South J, Woodall J, Stansfield J, Mapplethorpe T, Passey A, Bagnall AM. A qualitative synthesis of practice-based learning from case studies on COVID community champion programmes in England, UK. BMC Public Health. 2024 Jan 2;24(1):7. | Community-based volunteering supports outbreak management by extending reach into at-risk communities. This paper examines the application of a ‘community champions’ model in England, UK, during the COVID-19 pandemic. Evidence pre-pandemic shows that community champion interventions tap into social networks to strengthen connections with disadvantaged communities. During the pandemic, the UK government set up a COVID community champions funding award scheme for local authorities to develop local programmes that addressed emerging inequalities. The study aim was to identify transferable learning on community engagement in the pandemic by undertaking a secondary qualitative synthesis of practice-based case studies of local COVID community champion programmes. | Grade B |

**Demonstrating commitment to equity and inclusion in communication**

| **Reference** | **Abstract** | **GRADE** |
| --- | --- | --- |
| Adger WN, Hughes TP, Folke C, Carpenter SR, Rockstrom J. Social-ecological resilience to coastal disasters. Science. 2005 Aug 12;309(5737):1036-9. | Social and ecological vulnerability to disasters and outcomes of any particular extreme event are influenced by buildup or erosion of resilience both before and after disasters occur. Resilient social-ecological systems incorporate diverse mechanisms for living with, and learning from, change and unexpected shocks. Disaster management requires multilevel governance systems that can enhance the capacity to cope with uncertainty and surprise by mobilizing diverse sources of resilience. | Grade C |
| Cutter SL, Barnes L, Berry M, Burton C, Evans E, Tate E, Webb J. A place-based model for understanding community resilience to natural disasters. Global environmental change. 2008 Oct 1;18(4):598-606. | There is considerable research interest on the meaning and measurement of resilience from a variety of research perspectives including those from the hazards/disasters and global change communities. The identification of standards and metrics for measuring disaster resilience is one of the challenges faced by local, state, and federal agencies, especially in the United States. This paper provides a new framework, the disaster resilience of place (DROP) model, designed to improve comparative assessments of disaster resilience at the local or community level. A candidate set of variables for implementing the model are also presented as a first step towards its implementation. | Grade C |
| World Health Organization Strategic Communications Framework for Effective Communications, 2017. Geneva. https://www.who.int/docs/default-source/documents/communicating-for-health/communication-framework.pdf | WHO recognizes that effective, integrated and coordinated communication is integral to carrying out WHO’s goal to build a better, healthier future for people all over the world. The purpose of this Framework is to describe a strategic approach for effectively communicating WHO information, advice and guidance across a broad range of health issues: from chronic health issues to emerging and novel risks. WHO has made a significant investment to meet the growing need for information, advice and guidance, from increasing capacity to improved integration of available communication channels including media relations, social and online communications, branding, visual communications, and health and emergency risk communications. WHO needs all employees to understand and use communications effectively to achieve programmatic goals. This strategic approach is presented as a framework of principles for effective practice that apply to a broad range of communications functions. It reflects inputs and review by WHO communicators across WHO’s country, regional and headquarters offices. Tactics are included to develop communication products and activities that apply the principles. If these tactics and resources are used, communication at all levels of the Organization can be improved. The Framework is not designed as a communications strategy for particular diseases, specific health observances, or geographic regions. Instead, the principles and tactics can be used as resources to develop specific strategies to include communications that are more actionable, accessible, relevant, timely, understandable, and credible. It is a resource and reference designed for continuous updates as advised by all WHO staff involved in communications. | Grade A |
